# Elucidating the Role of Alkali‐Metal Cations in Nickel Oxide OER Catalysts Under Mild pH Conditions by Operando X‐Ray Absorption Spectroscopy

**DOI:** 10.1002/cssc.202502619

**Published:** 2026-04-17

**Authors:** Yu Age, Shun Tsunekawa, Arisu Sakai, Kazuki Harada, Ryohei Ishihara, Toshiaki Ina, Ke‐Hsuan Wang, Masaaki Yoshida

**Affiliations:** ^1^ Department of Applied Chemistry Yamaguchi University Yamaguchi Japan; ^2^ Department of Applied Chemistry Sanyo‐Onoda City University Yamaguchi Japan; ^3^ Japan Synchrotron Radiation Research Institute (JASRI) Sayo Japan

**Keywords:** alkali‐metal cation, electrocatalyst, nickel oxide catalyst, operando XAFS, water electrolysis

## Abstract

Understanding the role of alkali‐metal cations in oxygen evolution reaction (OER) catalysis is critical for designing efficient electrocatalysts that operate under mild pH conditions. In this study, we systematically investigate the effect of Li^+^, Na^+^, K^+^, and Cs^+^ ions on the OER activity of nickel oxide catalysts electrodeposited in pH 9.0 carbonate buffer. Electrochemical measurements reveal that the OER activity increases with increasing cation size, following the trend Li^+^ < Na^+^ < K^+^ < Cs^+^. In situ UV–vis spectroscopy shows that K^+^ and Cs^+^ promote the oxidation of Ni^2+^ to high‐valent Ni species (Ni^3+^/Ni^4+^) at lower potentials, indicating that ion diffusion and electrochemical reactions proceed more smoothly. Complementary operando Ni K‐edge and O K‐edge X‐ray absorption fine structure analyses confirm the structural transition from Ni(OH)_2_ (Ni^2+^) to catalytically active γ‐NiOOH (Ni^3.6+^) under OER conditions and highlight cation‐dependent differences in phase transition behavior and reversibility in the presence of K^+^ or Cs^+^. These findings demonstrate that K^+^ and Cs^+^ ions not only lower the Ni oxidation onset potential but also facilitate ion transport during water oxidation. This work provides valuable mechanistic insight into cation‐dependent OER enhancement and offers guidelines for the design of next‐generation electrocatalysts tailored for near‐neutral environments.

## Graphical abstract

Nickel oxide catalysts electrodeposited from carbonate buffer solutions containing various alkali‐metal cations (Li^+^, Na^+^, K^+^, Cs^+^) under mild pH conditions were investigated by operando X‐ray absorption fine structure (XAFS) analysis. The results reveal that although all catalysts converge to a common γ‐NiOOH active phase under OER conditions, K^+^ and Cs^+^ facilitate its formation and reversible stabilization, resulting in enhanced catalytic activity.

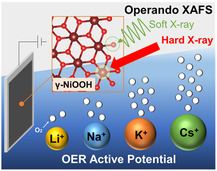

## 1 Introduction

Hydrogen production via water electrolysis using renewable energy sources such as solar power and wind power has attracted considerable attention [1, 2, 3, 4, 5]. However, in this process, the oxygen evolution reaction (OER) proceeds via a more complex reaction mechanism than the hydrogen evolution reaction (HER), resulting in a large overpotential that limits the overall energy efficiency of water splitting [6, 7, 8, 9]. Therefore, the development of a highly active OER electrocatalyst is required.

Noble‐metal oxides such as RuO_2_ and IrO_2_ have been reported to exhibit outstanding catalytic activity for the OER [10, 11, 12, 13]. Nevertheless, their limited availability and high cost restrict their suitability for large‐scale commercial applications. As a result, transition metal–based oxides, which are earth‐abundant and cost‐effective and exhibit high OER performance, have recently attracted interest as alternative catalyst materials [14, 15, 16, 17]. Ni‐based electrocatalysts have gained considerable attention because of their high activity and stability in alkaline environments [18, 19, 20, 21]. However, the use of alkaline media introduces concerns about environmental impact and corrosion [22, 23, 24]. By contrast, OER under neutral conditions is advantageous because it eliminates the need for expensive membranes or separators, reduces the environmental burden, and lowers the overall cost of water electrolysis [25, 26, 27]. Accordingly, increasing research efforts have been directed toward developing OER electrocatalysts that exhibit high performance under mild pH conditions [28, 29, 30, 31, 32, 33]. For example, Sun et al. reported that a Ni oxide catalyst (Ni–C_i_) electrodeposited in a carbonate solution at pH 8.3 demonstrated high OER activity [34]. Nocera et al. investigated a Ni oxide catalyst (Ni–B_i_), electrodeposited in a borate buffer at pH 9.2, and used operando X‐ray absorption spectroscopy (XAFS) to analyze its local structure [35]. Their results revealed that anodic activation of the Ni–B_i_ catalyst increased the oxidation state of the Ni centers from Ni^3+^ to an average of Ni^3.6+^, suggesting the formation of Ni^4+^ species. In addition, the activated catalyst films were shown to consist of sheet‐like clusters composed of edge‐sharing NiO_6_ octahedra, which function as active sites that contribute to the enhanced OER performance.

Electrolyte anions and cations are well known to strongly influence the OER activity of many transition metal oxide catalysts [36, 37, 38, 39, 40, 41]. Among them, nickel oxyhydroxide (NiOOH) possesses a layered structure composed of stacked NiO_6_ octahedra. Intercalation of alkali‐metal cations (Li^+^, Na^+^, K^+^, Cs^+^) into the interlayer gallery induces a NiOOH phase transition, elongation of the Ni—O bonds, and surface deprotonation, all of which have been discussed as key factors governing the OER performance of NiOOH [42, 43, 44]. These structural changes can be directly monitored by Raman spectroscopy and surface analysis techniques. For example, Garcia et al. reported that the Ni—O bending and stretching modes shift to lower wavenumbers in the Raman spectra of NiOOH in Cs^+^‐containing electrolytes, suggesting bond elongation and the formation of a superoxo intermediate (Ni—OO^−^) [45]. This intermediate is considered a precursor to O_2_ evolution and has been proposed to be stabilized by specific cation interactions. However, the apparent cation dependence cannot always be rationalized on the sole basis of ion size or direct ion–surface interactions; indirect factors such as electrolyte pH and basicity also play decisive roles. Görlin et al. showed by X‐ray absorption fine structure (XAFS) combined with density functional theory (DFT) that variations in OER activity correlate more closely with differences in electrolyte pH than with the identity of the alkali‐metal cation itself [46]. Consequently, a rigorous evaluation of cation effects requires experiments performed at a fixed pH. Notably, all of the previous related studies on cation effects have been conducted under strongly alkaline conditions (typically pH ≈ 13 or higher).

Therefore, the aim of the present study is to elucidate howalkali‐metal cations (Li^+^, Na^+^, K^+^, Cs^+^) influence the OER activity of nickel oxide catalysts under mild pH conditions (pH = 9.0) in a carbonate buffer. Strictly fixing the pH at 9.0 eliminates pH‐related effects, enabling the intrinsic effects of the cations to be examined independently. The investigation focuses on how thesecations affect the oxidation state and local structure of Ni species, as well as how they affect the chemical state of O species formed during the OER. To probe these effects, we conducted operando XAFS measurements using both hard X‐rays (>5 keV) and soft X‐rays (<2 keV) [47, 48, 49]. Simultaneous‐sis of Ni and O enables a qualitative identification of the true active species present at OER potentials [50, 51, 52]. Through this approach, we seek to provide new insights into the role of alkali‐metal cations in optimizing the OER activity of nickel oxide catalysts and to contribute to the development of more efficient water electrolysis processes.

## 2 Experimental Section

Nickel oxide catalysts (Ni–*M*C_i_, *M* = Li, Na, K, Cs; C_i_ denotes carbonate buffer) were electrodeposited as thin films on working electrodes by electro‐oxidative deposition at 1.7 V vs. RHE in a carbonate buffer (pH 9.0) containing 0.4 mM Ni(NO_3_)_2_ · 6H_2_O. After deposition, the electrolyte was replaced at 1.7 V with a Ni‐free carbonate buffer containing the same alkali‐metal cation to avoid cation cross‐contamination. A single alkali‐metal cation was used throughout electrodeposition, electrochemical measurements, and in situ/operando spectroscopy. Catalyst loading was controlled by adjusting the deposition time so that the absorbance at 440 nm reached 1.0 in in situ UV–vis spectra (Figure S1). Electrochemical measurements and in situ/operando analyses were conducted in Ni‐free 0.1 M *M*C_i_ electrolyte (pH 9.0). During electrolyte exchange, the electrode potential was maintained at 1.7 V and then lowered to 1.2 V for measurements. The catalysts were never exposed to air, and no electrochemical activation treatment was applied. Unless otherwise noted, measurements were performed on freshly prepared electrodes. Further experimental details are provided in the Supporting Information.

## 3 Results and Discussion

### 3.1 Characterization and Electrochemical Properties

Trace Fe impurities in electrolytes are known to influence the OER activity of Ni‐based catalysts [53]. In this study, the *M*C_i_ electrolytes were not Fe‐purified; therefore, Fe concentrations were quantified by ICP‐OES (Figure S2). All electrolytes contained Fe at similarly low levels in the ppb range, which is approximately three orders of magnitude lower than Fe concentrations typically reported to significantly affect Ni‐based OER activity [54]. Accordingly, while the contribution of Fe impurities cannot be completely excluded, Fe contamination is unlikely to be the primary factor governing the relative activity trends among different alkali‐metal cations in this work.

The Ni‐*M*C_i_ catalysts were characterized primarily in the as‐prepared state. SEM observations revealed that all Ni‐*M*C_i_ samples exhibit similar nanoscale network‐like morphologies (Figure S3). EDX, elemental mapping, and XPS analyses confirmed that the catalysts consist of Ni, O, C, and the corresponding alkali‐metal cations, which are homogeneously distributed without obvious phase segregation (Figures S4–S8). XRD patterns showed no detectable diffraction peaks other than those from the ITO substrate, indicating an amorphous structure (Figure S9). Ex situ Raman spectroscopy further confirmed the formation of NiOOH across all cations (Figure S10) [44, 55]. Taken together, these results indicate that the Ni‐*M*C_i_ catalysts possess an amorphous NiOOH‐like structure with the presence of alkali‐metal cations.

A key feature of this study is that the catalysts were handled without any exposure to air during electrochemical testing and operando measurements. Ex situ characterization requires handling outside this no‐air protocol; therefore, strict comparisons between ex situ data and the in situ condition require caution.

The catalytic activity of the Ni‐*M*C_i_ electrodes was investigated in pH 9.0 carbonate buffer electrolyte solution containing the same alkali‐metal cation (*M*C_i_). The Ni‐*M*C_i_ electrodes were electrodeposited onto a Au rotating disk electrode (RDE) to suppress the influence of the diffusion limitations in electrolyte solution and to improve the contact between the Ni‐*M*C_i_ catalyst and the *M*C_i_ electrolyte solution.

**FIGURE 1:**
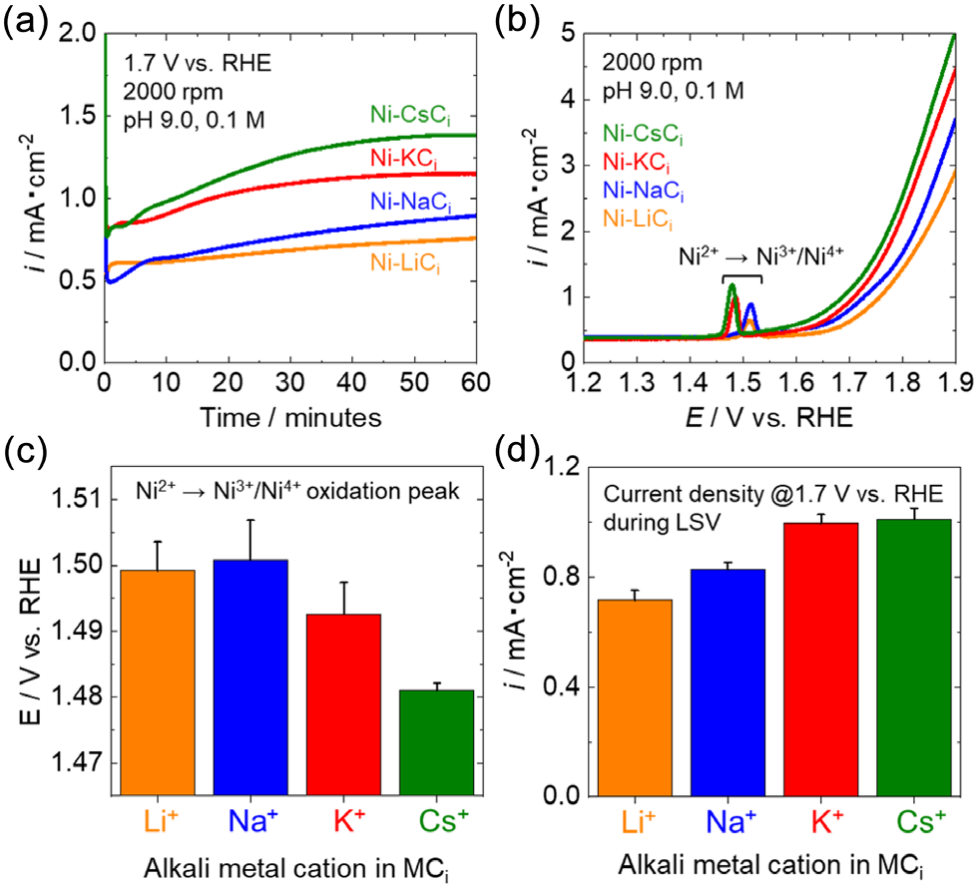
(a) Current–time measurements for the prepared Ni‐*M*C_i_ electrodes on a Au RDE at 1.7 V vs. RHE in *M*C_i_ at pH 9.0. (b) Current–voltage characteristics for the prepared Ni‐*M*C_i_ electrodes on a Au RDE at 1.7 V vs. RHE in *M*C_i_ at pH 9.0 (scan rate, 1 mV/s). In both graphs, the RDE rotation speed was set to 2000 rpm. The orange, blue, red, and green lines represent Ni‐LiC_i_, Ni‐NaC_i_, Ni‐KC_i_, and Ni‐CsC_i_ electrodes, respectively. (c) The Ni oxidation peak positions (Ni^2+^ → Ni^3.6+^) obtained from linear‐sweep voltammograms recorded at a scan speed of 1 mV/s. (d) The current density at 1.7 V obtained from linear‐sweep voltammograms at 1 mV/s. Error bars show the standard error from five measurements.

Figure 1a shows current–time measurements of the Ni‐*M*C_i_ electrodes on the Au RDEs, which were rotated at 2000 rpm, while a potential of 1.7 V was applied. This result shows that the water oxidation current remained stable for at least 60 min irrespective of the type of alkali‐metal cation and that the current density increased in the order Li^+^ < Na^+^ < K^+^ < Cs^+^. Figure 1b shows the LSV measurements of Ni‐*M*C_i_ electrodes recorded at a rotation speed of 2000 rpm. Following the observation of a current peak at ∼1.5 V, which was attributed to the oxidation of Ni (Ni^2+^ to Ni^3+^/Ni^4+^), increases in current associated with water oxidation were observed. The overpotential for OER followed the order Cs^+^ < K^+^ < Na^+^ < Li^+^. These results indicated that the OER activity of the Ni‐*M*C_i_ electrode increased with increasing ionic radius of the alkali‐metal cation, following the order Li^+^ < Na^+^ < K^+^ < Cs^+^. In addition, similar results were obtained even in the electrolyte solution with different pH values (Figure S11). This trend is consistent with previous studies; however, in the present study, substantially higher OER activity was observed in the presence of K^+^ and Cs^+^ compared with Li^+^ and Na^+^ [43, 44, 45]. The peak potential corresponding to the Ni oxidation tended to shift to lower values in the presence of K^+^ or Cs^+^ compared with the values in the presence of Li^+^ or Na^+^ (Figure 1c). The current density trend at 1.7 V was also higher in the presence of K^+^ or Cs^+^ than in the presence of Li^+^ or Na^+^ (Figure 1d). These results suggest that Ni oxidation proceeds at lower potentials when K^+^ or Cs^+^ is present, thereby promoting the formation of active species for water electrolysis and resulting in a reduced OER overpotential. Consistent with this interpretation, scan rate–dependent cyclic voltammetry (1–3 mV/s) showed that Ni‐CsC_i_ in CsC_i_ exhibits sharper Ni redox features with less scan rate–induced peak broadening than Ni‐LiC_i_ in LiC_i_, indicating more facile Ni oxidation/reduction kinetics (Figure S12). Furthermore, electrolyte cation‐switching experiments between LiC_i_ and KC_i_ at 1.8 V demonstrated that both Ni‐LiC_i_ and Ni‐KC_i_ electrodes exhibit higher OER activity in KCi and lower activity in LiC_i_ (Figure S13). These results indicate that the observed activity trend is governed primarily by the electrolyte cation rather than by the initial electrode state.

The Ni‐KC_i_ electrode showed a Tafel slope of 106 mV/dec when its catalytic activity was evaluated in 0.5 M KC_i_ (Figure S14). In a previous study, the Tafel slope for the OER on pure NiOOH catalysts in borate buffer solution (pH 9.2) was reported to range between 90 and 120 mV/dec, indicating that this catalyst exhibits favorable activity as a Ni‐based catalyst under mild pH conditions [56]. In addition, long‐term current–time measurements of Ni‐KC_i_ deposited on an ITO substrate at 1.7 V vs. RHE in 0.1 M KC_i_ (pH 9.0) confirmed stable OER activity for over 50 h (Figure S15). To confirm oxygen generation at the Ni‐LiC_i_ electrode, we conducted rotating ring‐disk electrode (RRDE) measurements (Figure S16).

### 3.2 In Situ UV–Vis/XAFS Spectroscopic Analysis

**FIGURE 2:**
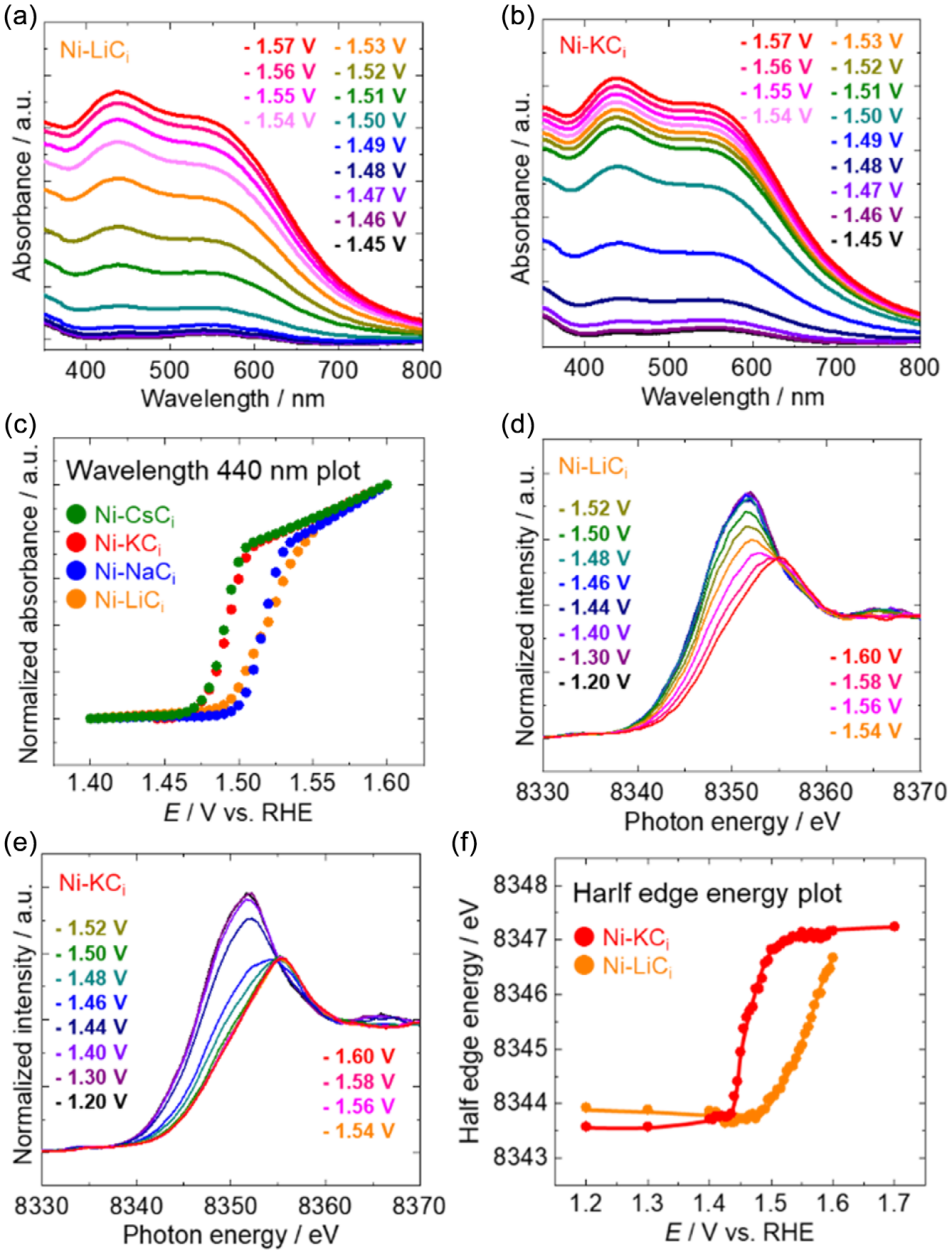
In situ UV–vis spectra of Ni‐*M*C_i_ in *M*C_i_ (pH 9.0): (a) Ni‐LiC_i_ and (b) Ni‐KC_i_. (c) Absorbance–potential plot at 440 nm in UV–vis spectra (normalized using absorbance values of 0 at 1.400 and 1 at 1.600 V). In situ Ni K‐edge XANES spectra of Ni‐*M*C_i_ in *M*C_i_ (pH 9.0): (d) Ni‐LiC_i_ and (e) Ni‐KC_i_. (f) Half‐edge energy‐potential plot obtained from normalized XAFS spectra.

In situ UV–vis measurements were performed to examine how alkali‐metal cations influence the potential‐dependent oxidation of Ni species. The applied potential was stepped from 1.400 to 1.600 V in 5 mV increments, during which Ni‐*M*C_i_ electrodes in *M*C_i_ (pH 9.0) showed a pronounced increase in absorbance between 1.47 and 1.56 V (Figures 2a,b, and S9). A normalized plot of the 440 nm absorbance (set to 0 at 1.400 and 1 at 1.600 V) demonstrates that Ni oxidation begins at lower potentials in K^+^ and Cs^+^‐containing electrolytes than in Li^+^ or Na^+^ (Figure 2c). To verify that the UV–vis response corresponds to Ni oxidation, in situ Ni K‐edge X‐ray absorption near edge structure (XANES) measurements were performed under identical electrochemical conditions. For both Li^+^ and K^+^, the absorption edge shifted to higher energies as the potential approached the oxidation onset (Figure 2d,e), indicating an increase in the Ni oxidation state. The half‐edge energy plot confirms that the oxidation onset occurs at a lower potential with K^+^ than with Li^+^ (Figure 2f). The potential‐dependent spectral evolution also shows that Ni oxidation proceeds more rapidly in the presence of K^+^. Overall, these results indicate that K^+^ and Cs^+^ promote the formation of higher‐valent Ni species (Ni^3+^/Ni^4+^) at lower potentials, thereby facilitating the generation of active OER intermediates and explaining the enhanced OER activity observed in K^+^ and Cs^+^ electrolytes.

### 3.3 Operando Ni K‐Edge XAFS

**FIGURE 3:**
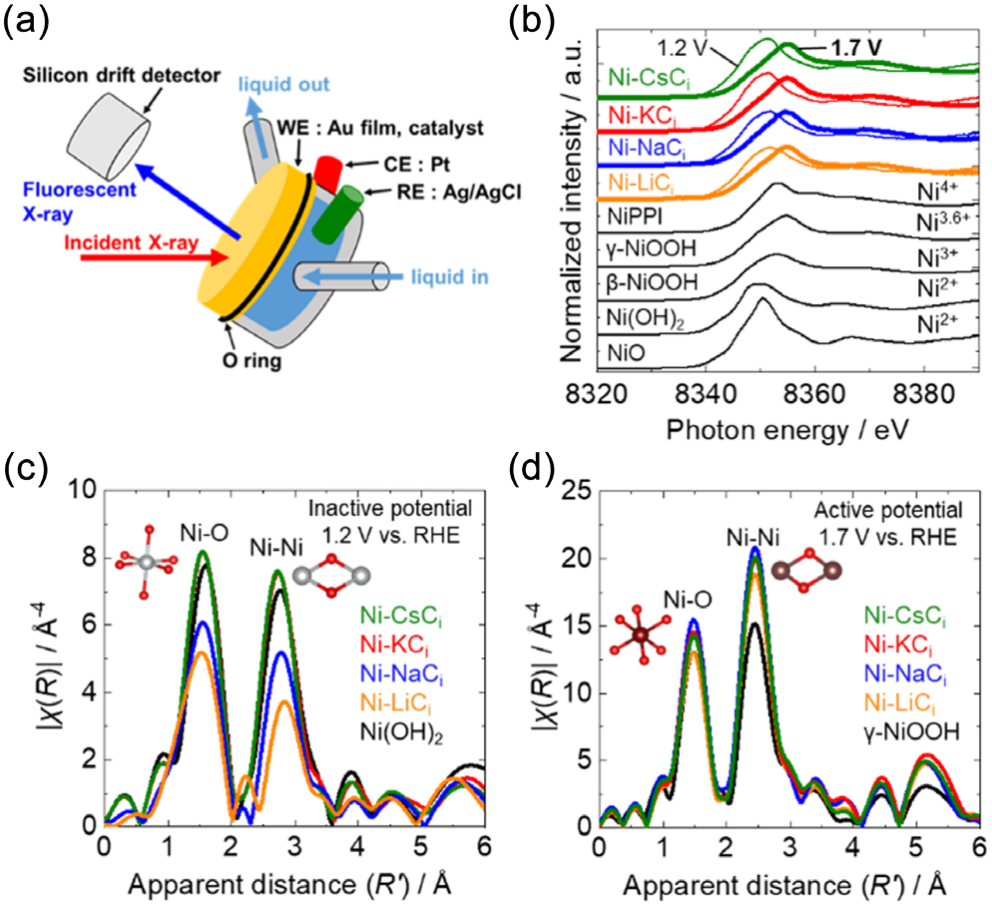
(a) Schematic of operando Ni K‐edge XAFS measurements in fluorescence mode under hard X‐rays. (b) Operando Ni K‐edge XAFS spectra of Ni‐*M*C_i_ at inactive (1.2 V vs. RHE, thin line) and active (1.7 V vs. RHE, thick line) potentials in *M*C_i_ at pH 9.0. (c) *k* ^3^‐weighted Ni K‐edge FT‐EXAFS analysis (3.0 ≤ *k* ≤  9.3, Hanning window function, not phase‐shift corrected) of the Ni‐*M*C_i_ catalyst at the inactive potential (1.2 V vs. RHE). (d) *k* ^3^‐weighted Ni K‐edge FT‐EXAFS analysis (3.0 ≤ *k* ≤ 11.0, Hanning window function, not phase‐shift corrected) of the Ni‐*M*C_i_ catalyst at the active potential (1.7 V vs. RHE). The spectra of Ni‐*M*C_i_ are represented as follows: orange for Ni‐LiC_i_, blue for Ni‐NaC_i_, red for Ni‐KC_i_, green for Ni‐CsC_i_. The spectra of Ni reference compounds are shown as black lines.

Operando Ni K‐edge XAFS measurements were conducted for Ni‐*M*C_i_ in *M*C_i_, under both an OER‐inactive potential (1.2 V) and an OER‐active potential (1.7 V). The experimental setup using hard X‐rays is illustrated in Figure 3a. The reference compounds were NiO (Ni^2+^), Ni(OH)_2_ (Ni^2+^), β‐NiOOH (Ni^3+^), γ‐NiOOH (Ni^3.6+^), and NiPPI [K_2_Ni(H_2_IO_6_)_2_] (Ni^4+^). The nominal oxidation states are assigned on the basis of previous reports [35]. A comparison of the absorption edge positions in the XAFS spectra revealed a clear positive shift for all of the Ni‐*M*C_i_ when the applied potential was increased from 1.2 to 1.7 V (Figure 3b), indicating an increase in the Ni oxidation state. The spectra acquired at an applied potential of 1.2 V are broadly consistent with that of the Ni(OH)_2_ reference, whereas the spectra acquired at 1.7 V well match that of the γ‐NiOOH reference. These results indicate that the Ni species are predominantly in a Ni(OH)_2_‐like, near‐Ni^2+^ state under the inactive potential and are oxidized to a γ‐NiOOH‐like state (approximately Ni^3.6+^) under the active potential. Further analysis based on the half‐absorption edge positions indicates that the average Ni valence at 1.2 V depends strongly on the alkali‐metal cation, with estimated values of 2.5 for Ni‐LiC_i_, 2.4 for Ni‐NaC_i_, 2.1 for Ni‐KC_i_, and 2.0 for Ni‐CsC_i_ (Figure S18). These results suggest that Ni reduction is relatively incomplete in the presence of Li^+^ and Na^+^, whereas Ni is reduced closer to the Ni^2+^ state in the presence of K^+^ and Cs^+^ under the inactive potential. This incomplete reduction under Li^+^/Na^+^ implies a less well‐defined redox baseline state prior to OER onset, which may hinder the efficient buildup of the catalytically competent γ‐NiOOH phase upon anodic polarization. In contrast, the average Ni valence at 1.7 V converged to 3.7 for Ni‐LiC_i_ and 3.9 for Ni‐NaC_i_, Ni‐KC_i_, and Ni‐CsC_i_, consistent with the formation of a highly oxidized γ‐NiOOH‐like phase under OER conditions.

To investigate the local structures of Ni species, we analyzed *k* ^3^‐weighted extended X‐ray absorption fine structure (EXAFS) oscillations at the Ni K‐edge for both 1.2 and 1.7 V applied potentials; the Fourier transformed spectra (FT‐EXAFS) are shown in Figure 3c,d, respectively. The spectra corresponding to an applied potential of 1.2 V show good agreement with the Ni(OH)_2_ reference, whereas those corresponding to 1.7 V correspond well with γ‐NiOOH, indicating that γ‐NiOOH is the OER‐active phase at the active potential. Notably, the FT‐EXAFS spectra of Ni‐LiC_i_ and Ni‐NaC_i_ at 1.2 V show substantially weaker peaks corresponding to Ni–O and Ni–Ni interactions, suggesting distortion in the Ni(OH)_2_ structure. By contrast, the spectra of Ni‐KC_i_ and Ni‐CsC_i_ exhibit stronger and clearer peaks, indicating that the Ni(OH)_2_ structure is more stable in the presence of K^+^ and Cs^+^. Consistent with these FT‐EXAFS trends, the *k* ^3^‐weighted EXAFS oscillations (Figure S19) also show clear potential and cation‐dependent differences. All Ni‐*M*C_i_ samples exhibit oscillation features consistent with the γ‐NiOOH reference at 1.7 V. At 1.2 V, the oscillation features are generally consistent with the Ni(OH)_2_ reference; however, the oscillation amplitude around *k* = 7 Å^−1^ was markedly damped for Ni‐LiC_i_ and Ni‐NaC_i_ and was almost canceled in the presence of Li^+^ (Figure S19). Such pronounced damping reflects a weakened Ni–Ni contribution, suggesting that under Li^+^ at 1.2 V, the characteristic local order of Ni(OH)_2_ is not fully developed and becomes locally disordered. This interpretation is also consistent with the XANES‐based average valence estimation, which indicates relatively incomplete reduction for the Li^+^ and Na^+^ electrolytes at the inactive potential.

Further FT‐EXAFS curve‐fitting analysis was carried out using the Ni(OH)_2_ crystal structure as the model at 1.2 V, with the Ni coordination number (*N*) fixed at 6. The Debye–Waller factors (*σ* ^2^) for the Ni—O bond were determined to be 0.010 ± 0.001 for Na^+^ and 0.006 ± 0.001 for both K^+^ and Cs^+^. Those for the Ni–Ni path were 0.011 ± 0.001 for Na^+^ and 0.007 ± 0.001 for both K^+^ and Cs^+^ (Figure S20a, S20b). These results indicate that the Ni(OH)_2_ structures were more significantly distorted in the presence of Na^+^ than in the presence of K^+^ or Cs^+^. Furthermore, the markedly weakened Ni–Ni path at 1.2 V in the presence of Li^+^ made robust fitting with the Ni(OH)_2_ model difficult, suggesting that Li^+^ strongly promotes local disordering at the inactive potential. Similarly, curve fitting of the data acquired under an applied potential of 1.7 V was performed using the γ‐NiOOH crystal structure as the model, with the Ni coordination number fixed at 6 (Figures S20c, S20d). The fitting results show good agreement with the experimental data for all of the Ni‐*M*C_i_ at the active potential. As a representative example of the potential‐dependent change, the fitted parameters for Ni‐KC_i_ were compared using FT‐EXAFS spectra processed under identical conditions (3.0 ≤ *k* ≤ 9.3, Hanning window function, not phase‐shift corrected) (Figures S20e, S20f). The Ni—O and Ni–Ni distances decrease from ∼2.04 and ∼3.09 at 1.2 to ∼1.88–1.89 Å and ∼2.82 Å at 1.7 V, respectively. Thus, increasing the potential from 1.2 to 1.7 V shortens the Ni—O bond by ∼0.15–0.16 Å and the Ni–Ni distance by ∼0.27 Å, reflecting local structural contraction and reorganization associated with the phase transition from Ni(OH)_2_ to γ‐NiOOH. It should be noted that, although the usable *k*‐range and fitting parameters varied with applied potential, the convergence of the active‐state local Ni structure across different alkali‐metal cations is directly evident from the operando EXAFSspectra at OER‐relevant potentials (Figure S20) and does not rely on quantitative fitting parameters.

To evaluate the reversibility of the phase transition from the optical response, operando UV–vis spectra were recorded while repeatedly switching the potential between 1.2 and 1.7 V (Figure S21). At 1.7 V, both Ni‐LiC_i_ and Ni‐CsC_i_ show higher visible absorption than at 1.2 V, indicating the absorption bands associated with highly oxidized Ni (γ‐NiOOH‐like) under OER conditions. Upon returning to 1.2 V, however, the recovery behavior depends on the cation: Ni‐LiC_i_ exhibits a progressive increase in the 1.2 V absorbance with cycling, indicating incomplete recovery, whereas Ni‐CsC_i_ shows nearly overlapping spectra at 1.2 V between cycles, demonstrating reproducible recovery to the low‐absorbance state. The current–time responses further show that the current at 1.7 V is consistently larger for Ni‐CsC_i_ than for Ni‐LiC_i_, confirming that higher OER activity is maintained in the presence of Cs^+^.

Collectively, these findings demonstrate that Ni‐*M*C_i_ adopts a Ni(OH)_2_ structure at the inactive potential and transitions to a γ‐NiOOH structure under the active potential, with the latter functioning as the OER‐active catalyst. In addition, the type of alkali‐metal cation appears to influence the reversibility of the electrochemically induced Ni oxidation state, as supported by operando UV–vis spectroscopy (Figure S21). These results suggest that Li^+^ and Na^+^ are more likely to exhibit incomplete reduction and larger local distortion at the inactive potential, whereas K^+^ and Cs^+^ tend to promote smoother and more reversible phase transition behavior. In addition, all Ni‐*M*C_i_ exhibited a γ‐NiOOH phase at the active potential.

### 3.4 Operando O K‐Edge XAFS

**FIGURE 4:**
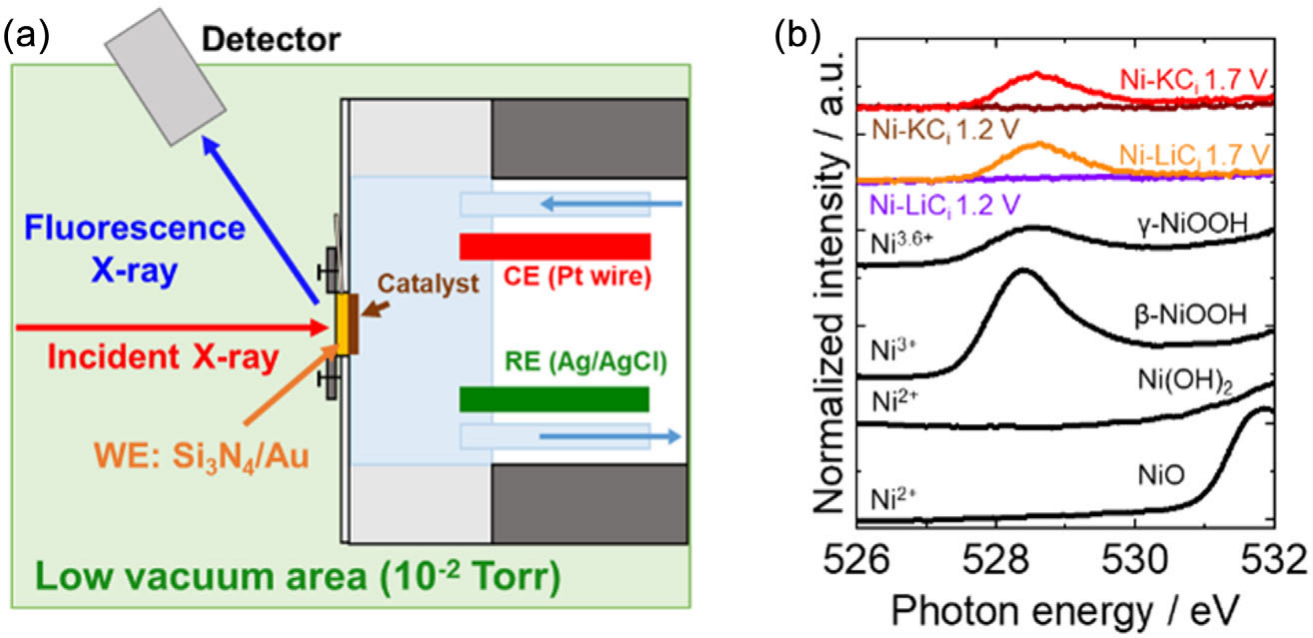
(a) Schematic of operando XAFS measurements in fluorescence mode under soft X‐rays. (b) Operando O K‐edge XAFS spectra of Ni‐LiC_i_ and Ni‐KC_i_ electrodes at inactive (1.2 V vs. RHE) and active (1.7 V vs. RHE) potentials in *M*C_i_ at pH 9.0. The purple and orange lines represent the Ni‐LiC_i_ electrode at 1.2 and 1.7 V, respectively; the brown and red lines represent the Ni‐KC_i_ electrode at 1.2 and 1.7 V, respectively. The black lines represent Ni oxide reference spectra.

To observe the structural changes from the perspective of the O species during the OER in Ni‐*M*C_i_, we conducted operando O K‐edge XAFS measurements using soft X‐rays. Figure 4a presents a schematic of the operando soft X‐ray O K‐edge XAFS measurements. Because soft X‐rays cannot penetrate air, measurements were performed under low‐vacuum conditions (10^−2^ Torr).

Figure 4b shows the O K‐edge XAFS spectra for Ni‐LiC_i_ and Ni‐KC_i_ during the OER at the inactive potential (1.2 V) and the active potential (1.7 V), along with reference spectra for Ni oxide compounds. A peak associated with an NiO_6_ structure was observed at approximately 528.7 eV. This peak is attributed to electron transitions to hybridized Ni(3d)–O(2p) states, corresponding to the e_g_ orbital symmetry and low‐spin d^7^ (Ni^3+^) and d^6^ (Ni^4+^) electron configurations, as previously reported [57]. In the spectra of Ni‐LiC_i_ or Ni‐KC_i_ under an applied potential of 1.2 V, no substantial peak was observed. However, in the spectra corresponding to 1.7 V, a peak appeared at approximately 528.7 eV. This result indicates that, at 1.2 V, the Ni species remain in their low oxidation state (Ni^2+^) with no observable electron transitions, whereas at 1.7 V, electron transitions occur, resulting in oxidation of the Ni to higher oxidation states (Ni^3.6+^).

A comparison of the O K‐edge XAFS spectra of Ni‐LiC_i_ and Ni‐KC_i_ with those of reference Ni oxides reveals that the spectra corresponding to 1.2 V closely resemble those of Ni(OH)_2_, whereas the spectra corresponding to 1.7 V align more closely with those of γ‐NiOOH. These results are consistent with the findings from the Ni K‐edge XAFS analysis regarding changes in the Ni valence and local structure and strongly support the conclusion that γ‐NiOOH serves as the OER‐active phase in Ni‐*M*C_i_.

**FIGURE 5:**
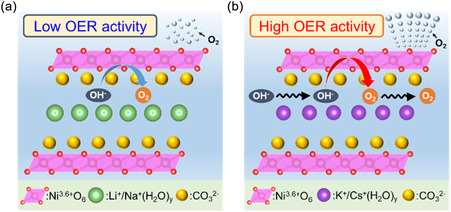
Schematic of the OER mechanism on the Ni‐*M*C_i_ under pH 9.0 conditions: (a) in Li^+^ or Na^+^ and (b) in K^+^ or Cs^+^. Carbonate anions reside near the surface of the catalyst layers, whereas alkali‐metal cations are hydrated and intercalated between the layers. The interlayer hydrated cations, K^+^(H_2_O)_*y*_ or Cs^+^(H_2_O)_*y*_, promote ion uptake into the interlayer of the Ni oxide catalyst, enhance OH^−^ transport, and thereby contribute to improved OER activity.

Figure 5 illustrates the proposed model for the OER process in Ni‐*M*C_i_. Taketsugu et al. reported a γ‐NiOOH structural model in which H_2_O molecules and K^+^ ions are intercalated between NiO_2_ layers [58], and Harada et al. showed that carbonate ions adsorb onto CoOOH surfaces [49]. The operando Cs L(III)‐edge XAFS spectra suggest that Cs^+^ ions exist in a hydrated form, Cs^+^(H_2_O)_*y*_, within the catalyst (Figure S22). The catalysts investigated in the present study consist of multilayered stacks of edge‐sharing NiO_6_ octahedral layers, feature intercalated hydrated alkali‐metal cations in the interlayer galleries, and likely host carbonate anions near the surface.

When the applied potential shifts from the inactive to the active state, the Ni species undergo a phase transition from Ni(OH)_2_ to γ‐NiOOH, accompanied by oxidation of Ni^2+^ to Ni^3.6+^. This oxidation proceeds via a stepwise proton‐coupled electron transfer (PCET) mechanism, in which the deprotonation of surface Ni—OH groups constitutes the rate‐determining step. As a result, under lower pH conditions, the onset potential of Ni oxidation is known to shift to more positive values [59, 60, 61, 62, 63]. By contrast, under the fixed pH of 9.0 used in the present study, in situ UV–vis/XANES revealed that high‐valent Ni species formed at lower potentials in the presence of K^+^ or Cs^+^ ions than in the presence of Li^+^ or Na^+^ ions. This cathodic shift is attributed to the presence of K^+^ and Cs^+^ cations, which are more weakly hydrated and more weakly solvated than Li^+^ and Na^+^. Owing to their less negative hydration enthalpies, lower charge density, and reduced solvation rigidity, these cations more readily relax the layered framework, provide electrostatic compensation, and facilitate ion uptake into the interlayer galleries [64, 65, 66]. As a consequence, Ni—OH deprotonation is facilitated and the delivery of OH^−^ ions to active sites is enhanced, thereby lowering the activation barrier of the PCET rate‐determining step and ultimately improving the OER activity.

Alternatively, K^+^ and Cs^+^ can facilitate ion diffusion and electrochemical reactions within the stacked layers, yielding a complementary route to the observed activity gains. Operando Ni K‐edge and O K‐edge XAFS measurements further support this interpretation: although local structural distortion of Ni(OH)_2_ was observed at inactive potentials in the presence of Li^+^ or Na^+^, the structure remained more stable under K^+^ or Cs^+^, indicating that K^+^ and Cs^+^ stabilize the layered framework and promote a smoother phase transition. Such intercalation and structural stabilization effects of alkali‐metal cations are consistent with the findings of Shehadeh et al., who demonstrated that alkali‐metal cations can be incorporated into the interlayer of Ni(Fe)OOH and thereby modulate its electronic structure and reaction intermediates [42]. In addition, the results of Gallenberger et al. revealed that increasing the cation size promotes the adoption of a more γ‐like NiOOH structure and facilitates water dissociation, further corroborating the beneficial role of K^+^ and Cs^+^ ions observed in the present work [43]. Moreover, Görlin et al. emphasized that the apparent cation dependence of OER activity is often strongly correlated with variations in electrolyte pH rather than the cation identity itself [46]. Thus, the control of the electrolyte at pH 9.0 in the present study excludes such pH‐related effects and enables the intrinsic role of alkali‐metal cations to be clearly identified. Importantly, electrolyte cation‐switching experiments (LiC_i_ ↔ KC_i_) at 1.8 V showed that both Ni‐LiC_i_ and Ni‐KC_i_ electrodes exhibit higher OER activity in KC_i_ and lower activity in LiC_i_, demonstrating that the activity trend is governed more strongly by the electrolyte cation than by the initial electrode state. Furthermore, operando Ni K‐edge and O K‐edge XAFS indicate that all Ni‐*M*C_i_ adopt γ‐NiOOH as the OER‐active phase at the active potential, with good agreement to the γ‐NiOOH model across the series. Taken together, these results suggest that the cation‐dependent activity differences are not primarily caused by geometric factors or by the formation of distinct active phases. Instead, they arise mainly from cation‐dependent modulation of access to and maintenance of the oxidized/deprotonated Ni state generated via PCET, while additional contributions from stabilization of oxygenated intermediates and interfacial transport effects cannot be excluded.

Regarding transport, Nakamura et al. proposed that cation hydration can generate an additional, cation‐dependent diffusional barrier layer, where the barrier increases with increasing rigidity of the first hydration shell (Li^+^ > Na^+^ > H^+^ > K^+^ > Cs^+^) [67]. Although this study addressed chloride diffusion under RRDE conditions, the underlying concept that hydration‐structured interfacial layers can modulate mass transport is general. Accordingly, in the present layered Ni oxyhydroxide system, hydrated K^+^ and Cs^+^ may mitigate transport penalties and facilitate ion motion within the stacked layers, providing additional rationale for the interpretation in Figure S5. Finally, mechanistic studies on Ni oxyhydroxides have identified a side‐on Ni‐OO‐Ni (superoxide‐like) intermediate under OER conditions [68]. Moreover, electrolyte alkali‐metal cations have been proposed to stabilize superoxo‐type intermediates via ion pairing (e.g., NiOO^−^−M^+^), with Cs^+^ providing stronger stabilization [45]. Such cation‐assisted stabilization offers a plausible link between hydrated K^+^ and Cs^+^ and the enhanced OER activity observed in this work.

### 3.5 Conclusion

In summary, we systematically investigated the effect of alkali‐metal cations (Li^+^, Na^+^, K^+^, Cs^+^) on Ni oxide electrocatalysts under near‐neutral conditions. K^+^ and Cs^+^ shifted the onset of Ni oxidation to lower potentials and enhanced the OER activity. In situ UV–vis/ XANES and operando Ni/O K‐edge XAFS analyses consistently revealed that these cations promote smoother structural transitions between Ni(OH)_2_ and the γ‐NiOOH states. Importantly, although all Ni‐*M*C_i_ catalysts converge to a common γ‐NiOOH active phase at OER potentials, the intrinsic γ‐NiOOH structure is essentially identical across the cation series. However, the kinetic accessibility and reversible stabilization of this oxidized state are strongly cation dependent. Therefore, the observed activity differences do not originate from structural differences in the final active phase itself, but rather from cation‐modulated phase transition dynamics and interfacial/interlayer ion transport that govern the formation and stabilization of the catalytically relevant Ni^3+^/Ni^4+^ state. These findings highlight the importance of cation‐dependent ion transport and phase transition dynamics in improving water oxidation catalysts, offering design principles for efficient electrocatalysts that operate in mild pH environments.

## Supporting Information

Additional supporting information can be found online in the Supporting Information section.

## Funding

This study was supported by Japan Science and Technology Corporation (JPMJGX23H2, JPMJSP2111); Japan Society for the Promotion of Science (25K01882, 22KJ2347, 23KJ1659, JPMJSP2111, JPMJSP2111); and Yamaguchi University.

## Conflicts of Interest

The authors declare no conflicts of interest.

## Supporting information

Supplementary Material

## Data Availability

The data that support the findings of this study are available from the corresponding author upon reasonable request.
