# Supplementary material for: Elucidating the Role of Alkali‐Metal Cations in Nickel Oxide OER Catalysts Under Mild pH Conditions by Operando X‐Ray Absorption Spectroscopy
Source: ChemSusChem. 2026 Apr 17;19(8):e202502619. doi: 10.1002/cssc.202502619 (PMC13089329; doi:10.1002/cssc.202502619)
Supplement: Supplementary file 1 — Supplementary Material [file CSSC-19-e202502619-s001.pdf]

SUPPORTING INFORMATION

Elucidating the Role of Alkali-Metal Cations in Nickel Oxide OER Catalysts under Mild pH Conditions by Operando X-ray Absorption Spectroscopy

Yu Age,[1] Shun Tsunekawa,[1] Arisu Sakai,[1] Kazuki Harada,[1] Ryohei Ishihara,[2] Toshiaki Ina,[3] Ke-Hsuan Wang,[2] and Masaaki Yoshida*[1]

[1] *Yamaguchi University, Tokiwadai, Ube, Yamaguchi 755-8611, Japan*

[2] *Sanyo-Onoda City University, 1-1-1, Sanyo-Onoda, Yamaguchi 756-0884, Japan*

[3] *Japan Synchrotron Radiation Research Institute (JASRI), Hyogo 679-5198, Japan*

E-mail: yoshida3@yamaguchi-u.ac.jp

Index

i) Experimental procedures S2

ii) *In-situ* UV-vis spectroscopy during electrodeposition S5

iii) Characterization (ICP, SEM, EDX, XPS, XRD, and Raman) S7

iv) Electrochemical measurements S16

v) *In-situ* UV-vis spectroscopic analysis S22

vi) Operando Ni K-edge XAFS S23

vii) Curve-fitting analysis of Ni K-edge FT-EXAFS spectra S25

viii) Operando UV-vis spectroscopic analysis S27

ix) Operando Cs L(III)-edge XAFS spectra S28

i) Experimental procedures

Sample preparation

Catalysts were prepared by electrodeposition using a potentiostat (Hokuto Denko, HA-151B) to control the electrode potential, a Pt wire counter electrode, and an Ag/AgCl (3.0 M NaCl) reference electrode. In all of the experiments, the electrode potential for the Ag/AgCl reference electrode was converted to that for the reversible hydrogen electrode (RHE) by adding 0.2 V + 0.059 V × pH to the measured potentials. A Au rotating disk electrode (RDE, ⌀4 mm), fluorine-doped tin oxide (FTO)-coated glass substrate (GEOMATEC), or indium tin oxide (ITO)-coated glass substrate (GEOMATEC) was used as the working electrode. Four aqueous solutions were prepared: 0.1 M lithium carbonate buffer (LiC_i aq), sodium carbonate buffer (NaC_i aq), potassium carbonate buffer (KC_i aq), cesium carbonate buffer (CsC_i aq), and ammonium carbonate buffer (NH_4C_i aq, as a control sample free of alkali-metal cations), all adjusted to pH 9.0. Milli-Q water (<5 ppb total organic carbon, resistivity > 18 MΩ·cm) was used in all of the solutions. The carbonate buffers (MC_i, $M = Li^+, Na^+, K^+, Cs^+$) were prepared by bubbling CO_2 gas into aqueous solutions of 0.1 M $LiOH\cdot H_2O$ (≥98.0%, FUJIFILM Wako Pure Chemical), 0.1 M NaOH (≥97.0%, Nacalai Tesque), 0.1 M KOH (≥85.0%, Nacalai Tesque), 0.1 M CsOH (≥99.0%, 50 wt% aqueous solution, Sigma-Aldrich), or 0.1 M NH_3 aq (28 wt%, Nacalai Tesque). Nickel oxide (Ni-MC_i, M = Li, Na, K, Cs) catalysts were prepared by electro-oxidative deposition of thin films onto the working electrodes at 1.7 V vs. RHE using a carbonate buffer containing 0.4 mM solution of $Ni(NO_3)_2\cdot 6H_2O$ (≥99.9%, FUJIFILM Wako Pure Chemical). Subsequently, the electrolyte was replaced with a Ni-free carbonate buffer solution containing the same cation while maintaining the electrode potential at 1.7 V vs. RHE. A single alkali-metal cation was used in the electrolyte throughout the entire workflow, from catalyst electrodeposition to electrochemical activity tests and in-situ/operando measurements, to avoid cross-contamination by other alkali-metal cations. The catalyst loading was normalized by adjusting the electrodeposition time such that the absorbance at 440 nm in the UV-vis spectrum reached 1.0 Abs.

Characterization

Fe impurities in the electrolyte were identified and quantified by inductively coupled plasma optical emission spectroscopy (ICP-OES; SPS3500). The surface structure, surface elemental composition, and crystal structure of Ni-MC_i were characterized by scanning electron microscopy (SEM; JEOL JSM-7600F), energy dispersive X-ray spectroscopy (EDX; JEOL JED-2300), X-ray photoelectron spectroscopy (XPS; Thermo

Scientific, K-Alpha), X-ray diffraction (XRD; Rigaku Ultima), and Raman spectroscopy (NRS-7100, JASCO).

Electrochemical measurements

Electrochemical measurements were conducted in Ni-free 0.1 M $M\mathrm{C_i}$ solution (pH 9.0) using a three-electrode configuration with a Pt wire as the counter electrode, an Ag/AgCl (3.0 M NaCl) electrode as the reference electrode, and a Au rotating disk electrode (RDE) as the working electrode. Constant-potential measurements and linear sweep voltammetry (LSV) were performed at a rotation speed of 2000 rpm using a rotating electrode apparatus (BAS, RRDE-3A), with the potential controlled by a potentiostat. Unless otherwise specified, all current densities were normalized to the geometric area of the Au disk electrode. Immediately after the replacing the deposition solution with the Ni-free electrolyte, the potential was lowered to 1.2 V vs. RHE to conduct electrochemical activity tests and *in-situ*/operando measurements. In all electrochemical and *in-situ*/operando measurements, the catalysts were never exposed to air, including during electrolyte exchange. In addition, no activation treatment (e.g., repeated oxidation-reduction cycling) was applied. This was done to keep the catalyst morphology as consistent as possible and to clearly decouple the Ni redox features from the OER response. Unless otherwise noted, the potential sweep from the inactive to the active region was performed only once, and measurements were carried out on freshly prepared catalysts.

***In-situ*/Operando UV–vis measurements**

In-situ difference UV–vis absorption spectra were obtained in diffuse transmission mode using a UV–vis spectrophotometer (V-650; JASCO) equipped with an integrating sphere (ISV-722; JASCO); the spectra were acquired using a previously reported method.[1-3] In this analysis, an ITO electrode enclosed in a Teflon electrochemical cell was aligned with the front of the integrating sphere for diffuse transmission measurements. The sample was maintained at a specific potential in $M\mathrm{C_i}$ at pH 9.0 during spectral acquisition. Difference spectra were obtained by recording spectra relative to that acquired under open-circuit conditions.

***In-situ*/Operando Ni K-edge and Cs L(III)-edge XAFS measurements**

In-situ/Operando Ni K-edge and Cs L(III)-edge XAFS spectra were acquired using a Si(111) double-crystal monochromator at beamline BL01B1 of SPring-8 (JASRI). For the

operando XAFS measurements, a Au-deposited film (Ac/PP) was prepared as the working electrode and was mounted in a Teflon electrochemical cell sealed with an O-ring. In this setup, the fluorescence XAFS spectra were acquired during the electrochemical reaction using a Si drift detector equipped with a Co filter and Soller slits to eliminate elastically scattered X-rays. Reference spectra for Ni compounds, including NiO, $Ni(OH)_2$, γ-NiOOH, β-NiOOH, and $K_2Ni(H_2IO_6)_2$ [potassium nickel(IV) paraperiodate, NiPPI], were previously measured in transmission mode at the Photon Factory (KEK). The raw data were energy-calibrated on the basis of the first maximum of the first derivative (8333.0 eV) of previously reported Ni foil spectra.[3-5] For the Cs L(III)-edge measurements, operando fluorescence spectra were acquired under the same electrochemical configuration and detector setup. The raw data were energy-calibrated on the basis of the first maximum of the first derivative (5011.3 eV) of a previously reported CsCl spectrum.[6] Reference spectra for Cs compounds, including $CsNO_3$, Cs_2SO_4, $CsNO_3$ aq, and Cs_2SO_4 aq, were previously measured in fluorescence mode at SPring-8. The raw XAFS spectra were analyzed using the ATHENA/ARTEMIS program, and three-dimensional crystal visualizations were performed using the VESTA program.[7-9]

Operando O K-edge XAFS measurements

Operando O K-edge XAFS measurements were performed in fluorescence mode at beamline BL16A of KEK-PF. A custom-built Teflon electrochemical cell was used. A Au-coated Si_3N_4 membrane electrode was placed in a vacuum chamber, and the incident X-ray beam passed through the Si_3N_4 window behind the Au electrode. Fluorescence signals were collected by a Si drift detector and normalized to the incident beam intensity. The photon energy of the O K-edge XAFS spectra was calibrated on the basis of the first peak of O_2 gas at 530.80 eV.[5] Note that the *ex situ* O K-edge XAFS measurements for reference compounds were conducted in total electron yield mode at beamline BL7A of KEK-PF.

ii) *In-situ* UV-vis spectroscopy during electrodeposition

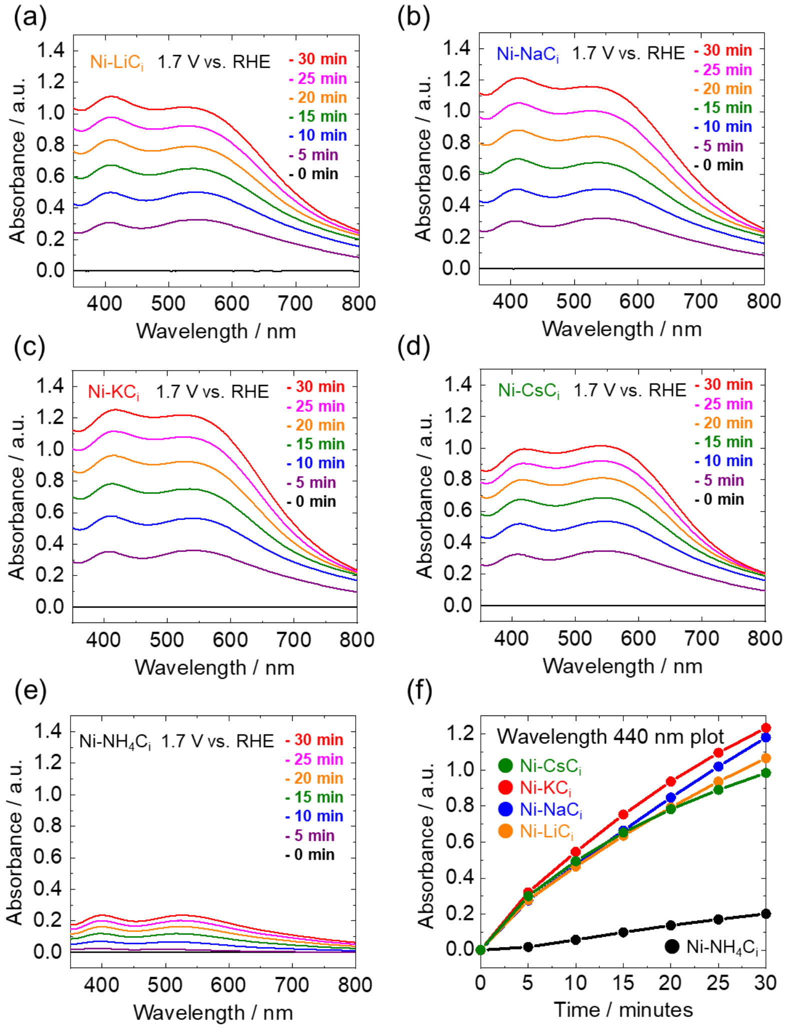

Figure S1. *In-situ* UV-vis spectra of (a) Ni-LiC$_i$, (b) Ni-NaC$_i$, (c) Ni-KC$_i$, (d) Ni-CsC$_i$, (e) Ni-NH$_4$C$_i$ during electrodeposition on ITO-coated glass substrates (Ni-NH$_4$C$_i$ is a non-alkali-metal cation sample). (f) Absorbance-time plot at 440 nm in UV-vis spectra. The background for the UV-vis spectra was collected at open circuit. During electrodeposition at 1.7 V vs. RHE, the samples containing alkali-metal cations (Li^+, Na^+, K^+, and Cs^+) exhibited a steady increase in the broad UV-vis absorption with time, and the absorbance at 440 nm increased almost monotonically. This behavior indicates continuous growth of the electrodeposited catalyst film on the ITO substrate. In contrast, Ni-NH$_4$C$_i$, which contains no alkali-metal cation, showed only a negligible increase in absorption, implying strongly suppressed film growth; therefore, it is difficult to normalize the catalyst films by achieving comparable film thickness and loading. Overall, these results demonstrate that the presence of alkali-metal cations substantially alters the electrodeposition behavior and promotes the formation of the catalyst film.

iii) Characterization (ICP, SEM, EDX, XPS, XRD and Raman)

Sample	Fe concentration (in ppb)
Milli-Q Water	1.1 (0.5)
pH 9.0 LiC_i aq	2.7 (0.9)
pH 9.0 NaC_i aq	4.3 (1.1)
pH 9.0 KC_i aq	3.3 (1.0)
pH 9.0 CsC_i aq	1.7 (0.6)

Figure S2. Fe concentration (ppb) in Milli-Q water and pH 9.0 MC_i aqueous electrolytes determined by ICP-OES using radial viewing. The sample uptake flow rate was 2 mL min^{-1}. Fe was monitored at 259.94 nm with background correction at $\pm 2.38 \times 10^{-2}$ nm (left and right). Each sample was measured three times independently, and the mean values are listed in the figure (values in parentheses indicate the standard deviation). Calibration curves were constructed using Fe standards at Blank, 0.1 ppm, 0.5 ppm, and 1 ppm. Note that the Fe concentrations in the sample solutions were extremely low, and thus the ICP-OES quantification involves a certain degree of uncertainty. Nevertheless, although the carbonate buffer used in this study was not purified, the level of Fe contamination was on the order of a few ppb, which is substantially lower than the Fe impurity levels reported to be problematic in previous studies. Moreover, the detected Fe contents were similarly low across all solutions, and no pronounced differences among the electrolytes were observed.

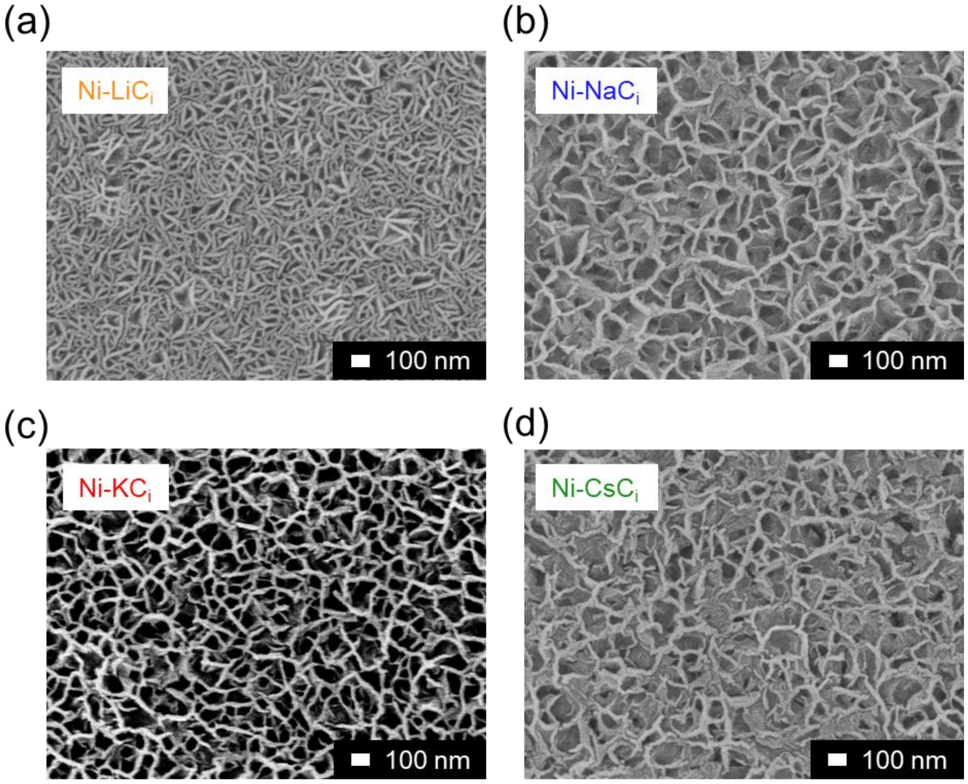

Figure S3. SEM images of (a) Ni-LiC$_i$, (b) Ni-NaC$_i$, (c) Ni-KC$_i$, and (d) Ni-CsC$_i$ electrodeposited onto FTO-coated glass substrates (as-prepared). A thin Pt film was deposited onto the surface of each sample to ensure electrical conductivity. These results indicate that the surface morphology of the catalysts is composed of a nanoscale network-like structure.

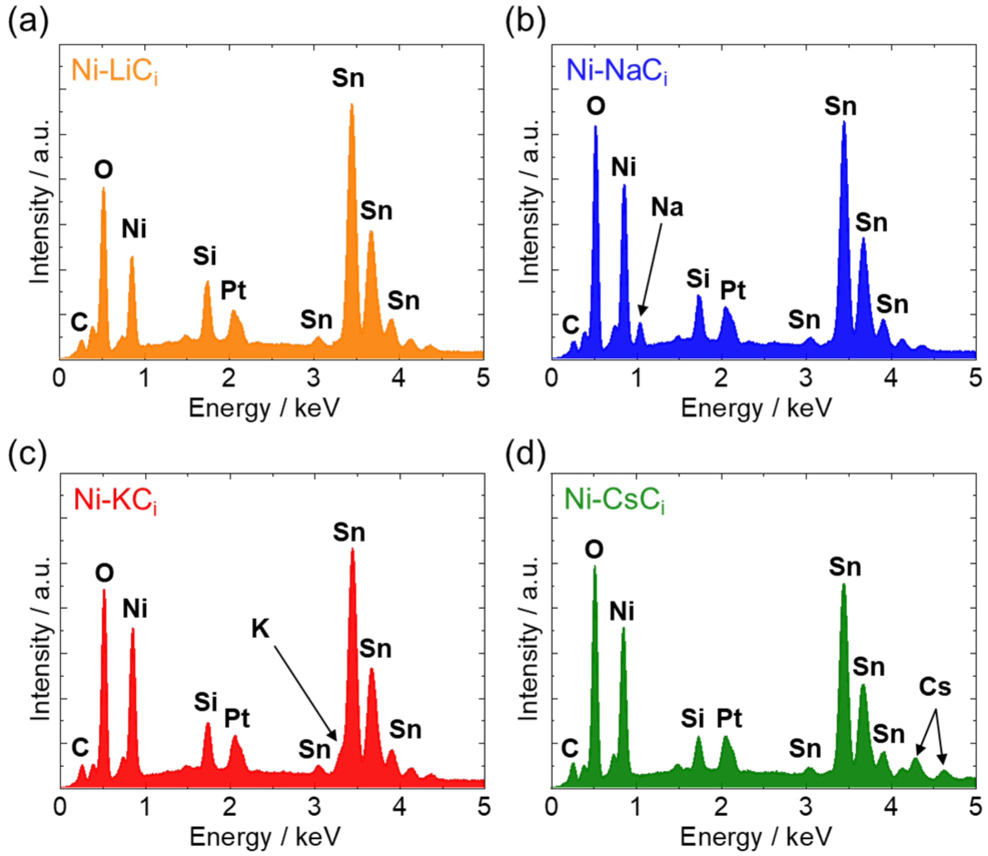

Figure S4. SEM–EDX spectra for (a) Ni-LiC_i, (b) Ni-NaC_i, (c) Ni-KC_i, and (d) Ni-CsC_i electrodeposited onto FTO-coated glass substrates (as-prepared). A thin Pt film was deposited onto the surface of each sample to ensure electrical conductivity. These results indicate that the catalysts consist of Ni, O, C, and alkali-metal cations.

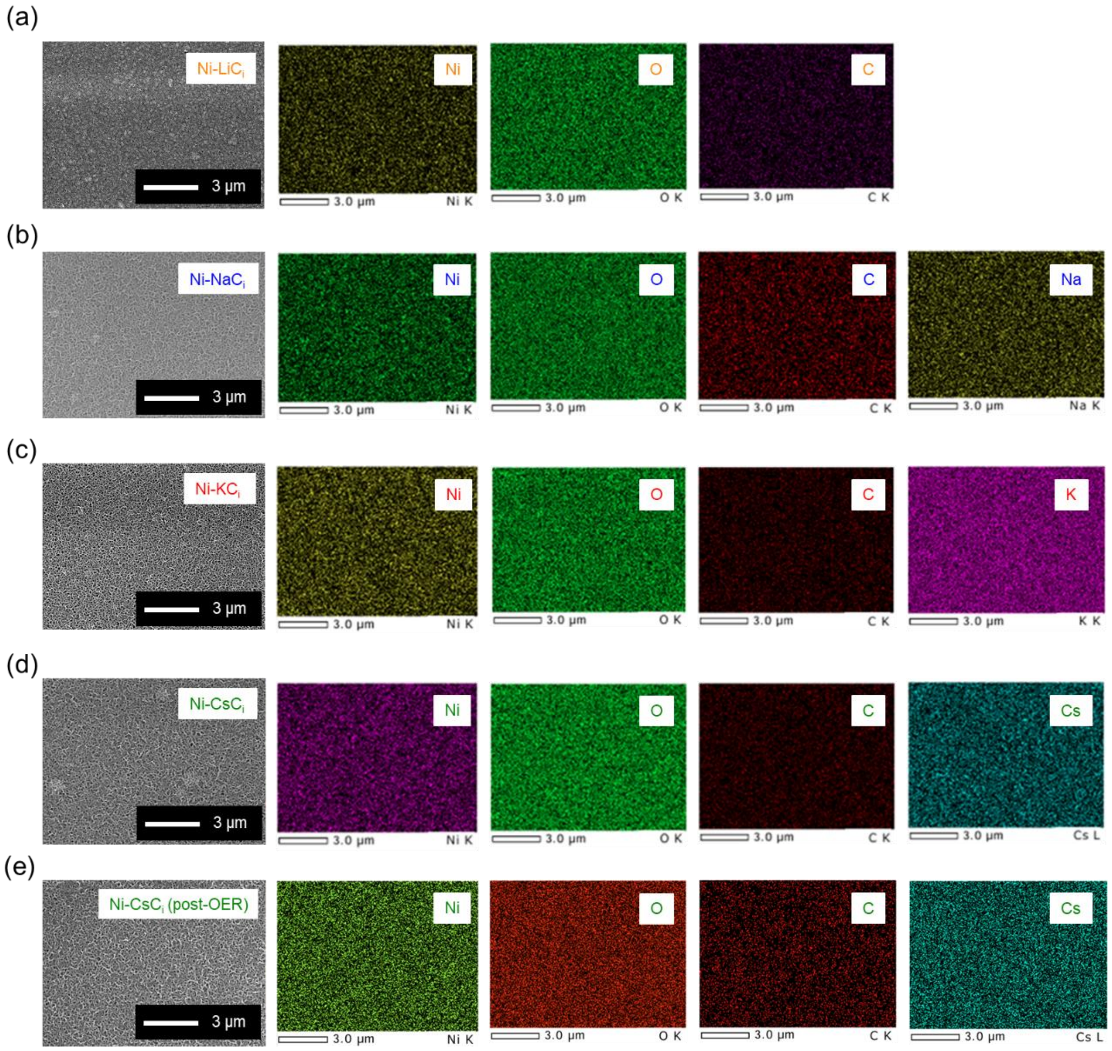

Figure S5. SEM images and EDX mappings of Ni, O, C, and the corresponding alkali metal cation (Na, K, or Cs) for Ni-*M*C_i electrodeposited on FTO-coated glass substrates: (a) Ni-LiC_i, (b) Ni-NaC_i, (c) Ni-KC_i, (d) Ni-CsC_i, (e) Ni-CsC_i after OER operation in CsC_i at 1.7 V for 10 h (post-OER). In both the as-prepared and post-OER samples, the elemental maps indicate a spatially uniform distribution of the detected elements across the catalyst film within the mapped area, with no obvious phase segregation. *Li could not be reliably detected by EDX and is therefore not shown.*

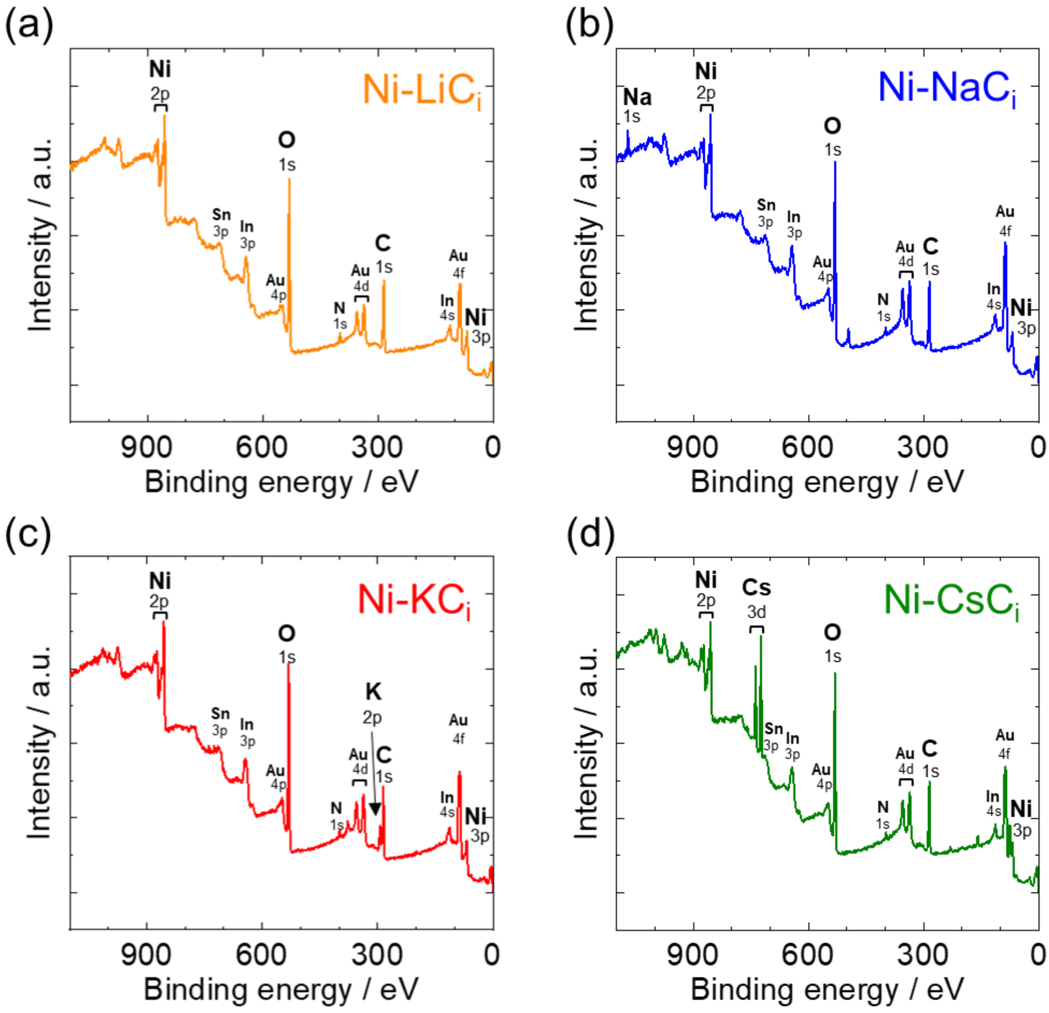

Figure S6. XPS spectra for (a) Ni-LiC$_i$, (b) Ni-NaC$_i$, (c) Ni-KC$_i$, and (d) Ni-CsC$_i$ electrodeposited onto ITO substrates (as-prepared). Au was sputtered onto the specimens to allow for the calibration of energy values. These results indicate that the catalysts are composed of Ni, O, C, and alkali-metal cations.

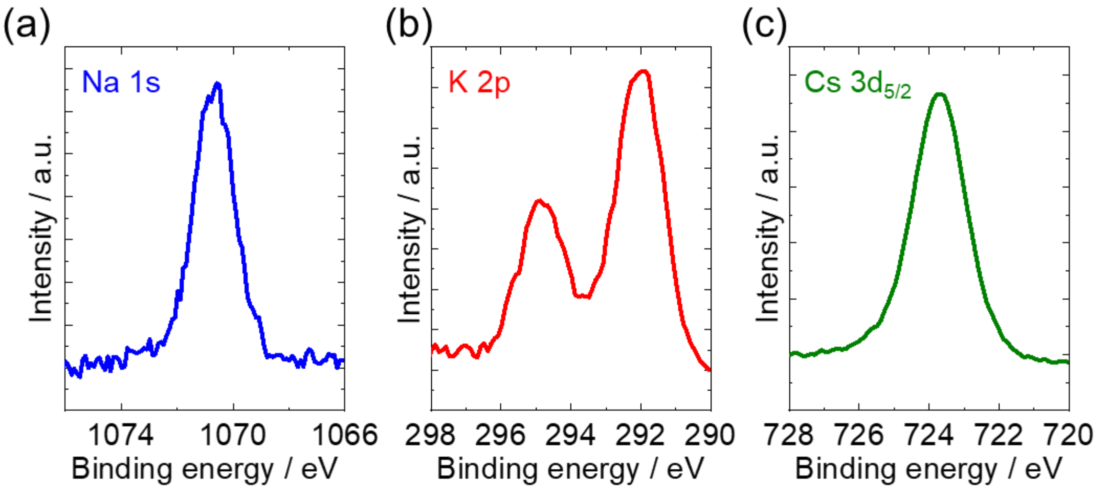

(d)

Ni-*M*C$_i$ (as-prepared)	c(Ni)	c(M^+)	c(M^+) / c(Ni)
Ni-LiC$_i$	14.56	-	-
Ni-NaC$_i$	15.34	2.68	0.17
Ni-KC$_i$	15.83	2.34	0.15
Ni-CsC$_i$	15.56	2.69	0.17

Figure S7. Core-level XPS spectra of alkali-metal cations on as-prepared Ni-*M*C$_i$: (a) Na 1s for Ni-NaC$_i$, (b) K 2p (doublet) for Ni-KC$_i$, and (c) Cs 3d$_{5/2}$ for Ni-CsC$_i$. (d) Surface atomic concentrations of Ni c(Ni) and alkali-metal cations c(M^+), and their ratio c(M^+)/c(Ni), estimated from the XPS peak areas using the Ni 2p, O 1s, C 1s, and Na 1s/K 2p/Cs 3d$_{5/2}$ signals. The exact concentration of Li^+ was not calculated as the core level spectrum hardly shows any intensity (Li also has a very low photoionization cross section). The c(M^+)/c(Ni) values for Na^+, K^+, and Cs^+ were comparable, suggesting similar surface loadings of alkali-metal cations among these catalysts.

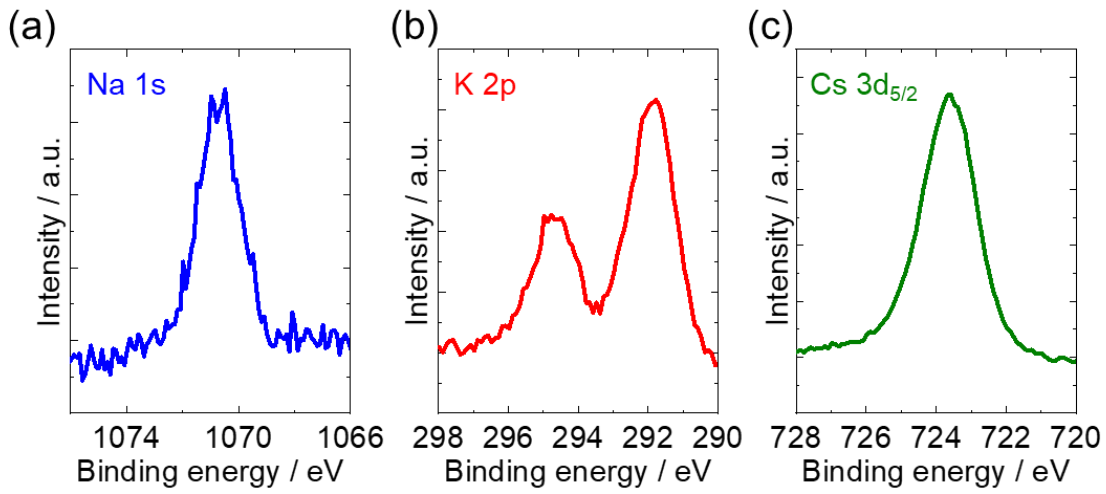

(d)

Ni-MC_i (post-OER)	c(Ni)	c(M^+)	c(M^+) / c(Ni)
Ni-NaC_i	15.26	1.57	0.10
Ni-KC_i	16.91	2.50	0.15
Ni-CsC_i	16.01	2.55	0.16

Figure S8. Core-level XPS spectra of alkali-metal cations on post-OER Ni-MC_i (after 10 h OER operation): (a) Na 1s for Ni-NaC_i, (b) K 2p (doublet) for Ni-KC_i, and (c) Cs $3d_{5/2}$ for Ni-CsC_i. (d) Surface atomic concentrations of Ni c(Ni) and alkali-metal cations c(M^+), and their ratio c(M^+)/c(Ni), estimated from the XPS peak areas using the Ni 2p, O 1s, C 1s, and Na 1s/K 2p/Cs $3d_{5/2}$ signals. The post-OER Ni-LiC_i sample was not analysed because Li was not detectable in the as-prepared sample. These results indicate that the alkali-metal cations are retained on the catalyst surface even after prolonged OER. Additionally, the c(Na^+)/c(Ni) ratio decreases after OER compared with the as-prepared sample (Figure S7.), suggesting a partial loss of Na species from the surface.

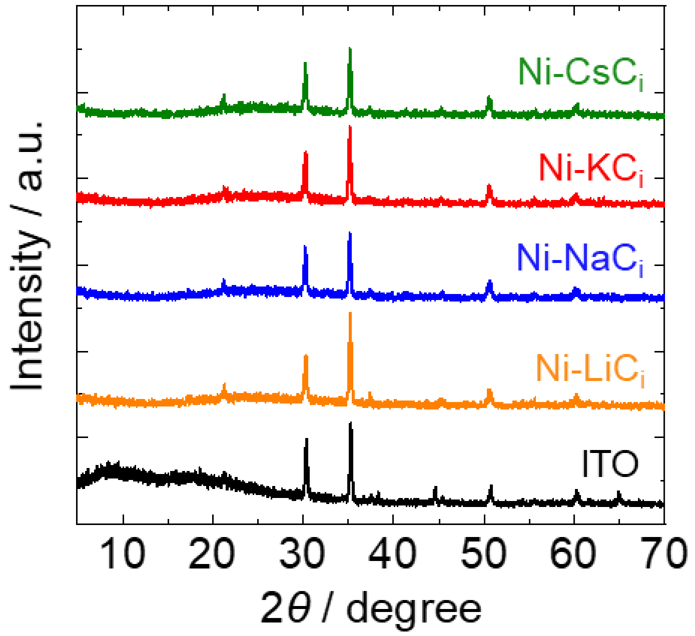

Figure S9. XRD patterns of the prepared Ni-LiC$_i$ (orange), Ni-NaC$_i$ (blue), Ni-KC$_i$ (red), and Ni-CsC$_i$ (green) electrodeposited onto ITO substrates (as-prepared), along with the pattern of the bare ITO substrate (black). XRD patterns derived from Ni-MC$_i$ (M = Li, Na, K, Cs) were not observed, suggesting that the structures of the Ni-MC$_i$ samples are amorphous.

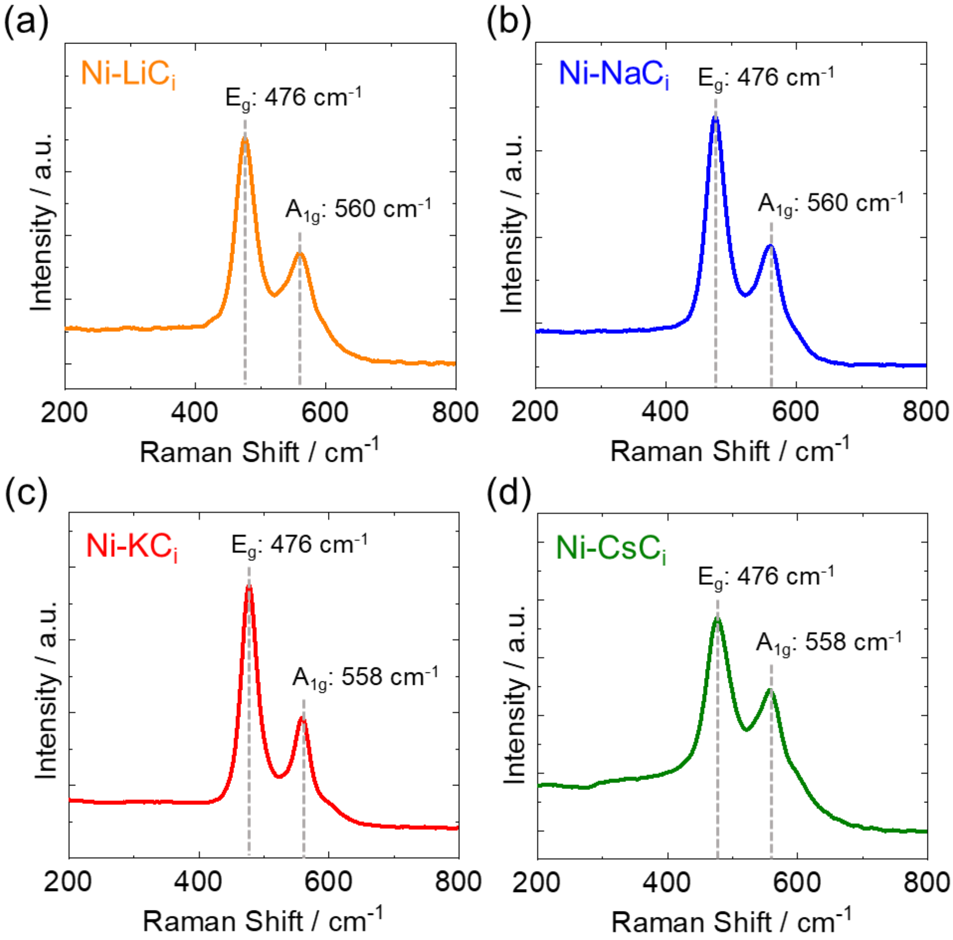

Figure S10. *Ex-situ* Raman spectra of the prepared (a) Ni-LiC$_i$ (orange), (b) Ni-NaC$_i$ (blue), (c) Ni-KC$_i$ (red), and (d) Ni-CsC$_i$ (green) electrodeposited onto ITO substrates. The Raman spectra show the characteristic NiOOH doublet at 476 and 558–560 cm^{-1} across all of the cations. These peak positions are consistent with those of canonical NiOOH; we therefore concluded that the catalyst films contain NiOOH, as confirmed by Raman characterization.[10-11]

iv) **Electrochemical measurements**

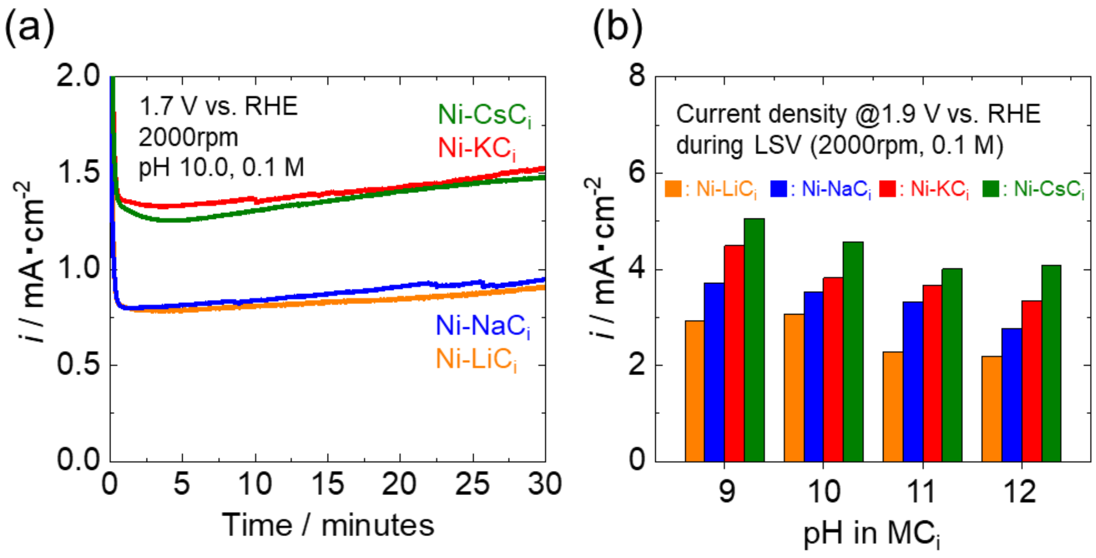

Figure S11. (a) Current–time measurements for the prepared Ni-MC_i electrodes on a Au RDE at 1.7 V vs. RHE in MC_i at pH 10.0. (b) Current–pH dependence at 1.9 V vs. RHE during current–voltage measurements (scan rate: 1 mV/s) of Ni-MC_i electrodes on a Au RDE in MC_i at pH 9, 10, 11, or 12. In both graphs, the RDE rotation speed was set to 2000 rpm. The orange, blue, red, and green lines correspond to Ni-LiC$_i$, Ni-NaC$_i$, Ni-KC$_i$, and Ni-CsC$_i$, respectively. These results indicate that Ni-KC$_i$ and Ni-CsC$_i$ exhibit greater catalytic activity than Ni-LiC$_i$ and Ni-NaC$_i$, even when the solution pH is varied.

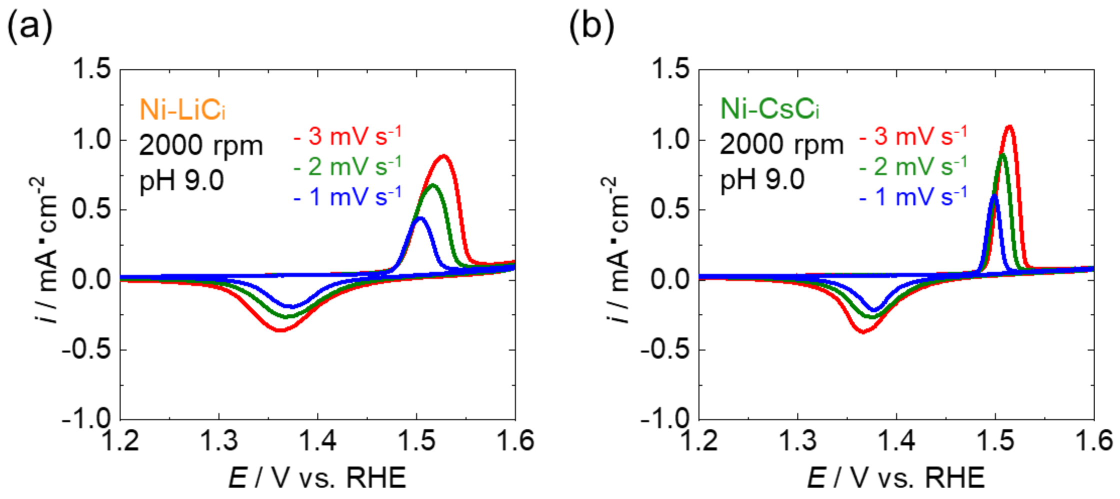

Figure S12. Cyclic voltammograms (CVs) of Ni-MC_i electrodes on a Au RDE in MC_i: (a) Ni-LiC_i and (b) Ni-CsC_i. The RDE rotation speed was set to 2000 rpm. The blue, green, and red lines correspond to scan rates of 1 mV s^{-1}, 2 mV s^{-1}, 3 mV s^{-1}, respectively. All CVs were recorded within 1.2-1.6 V vs. RHE. These results indicate that Ni-CsC_i exhibits relatively sharper redox features and a smaller extent of scan-rate-induced peak broadening than Ni-LiC_i, suggesting more facile Ni oxidation/reduction (a better ability to follow the potential sweep) in the presence of Cs^+, whereas the Ni redox process is less accessible under Li^+-containing conditions. The measurements were performed sequentially from higher to lower scan rates; therefore, the oxidation peak potential in the 1 mV s^{-1} CV is slightly shifted to higher potentials compared with that of a freshly prepared catalyst. Thus, the oxidation peak position at 1 mV s^{-1} in this figure should not be directly compared with that in Figure 1 of the main text.

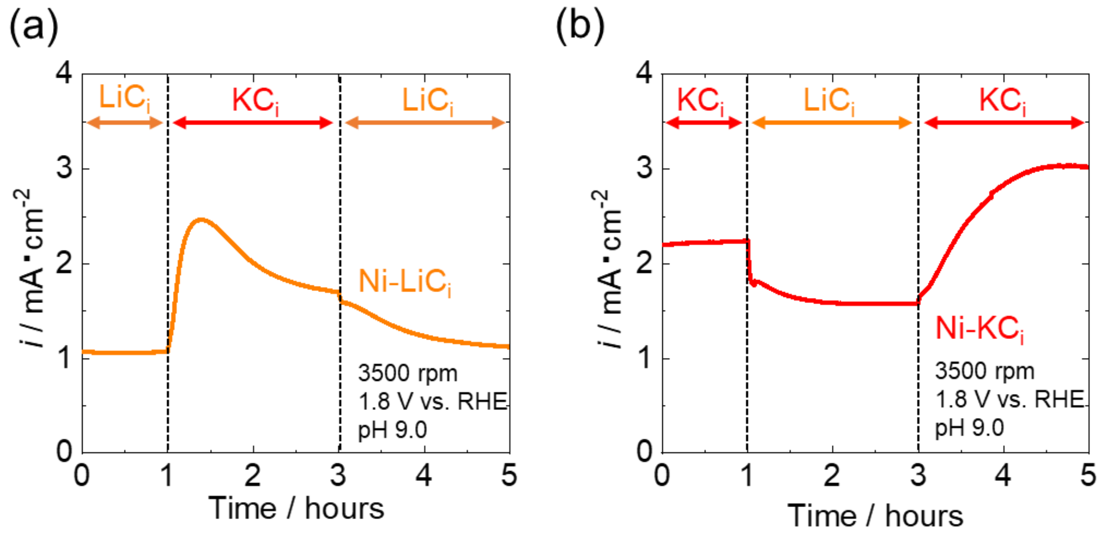

Figure S13. Current–time measurements of Ni-MC$_i$ electrodes during electrolyte cation switching at 1.8 V vs. RHE. (a) Ni-LiC$_i$ and (b) Ni-KC$_i$ electrodes were measured on a Au rotating disk electrode (RDE), while the electrolyte was exchanged between LiC$_i$ and KC$_i$ as indicated by the arrows. The rotation speed was fixed at 3500 rpm. The measurements were started after initiating the activity test of the Ni-MC$_i$ electrode in MC$_i$, after the current had stabilized at an approximately constant value (steady state). These results indicate that both the Ni-LiC$_i$ and Ni-KC$_i$ electrodes exhibit relatively higher activity in KC$_i$ and lower activity in LiC$_i$. Therefore, the current response depends more strongly on the alkali-metal cation in the electrolyte (Li^+/K^+) than on the initial electrode state, indicating that the cation effect is dominant.

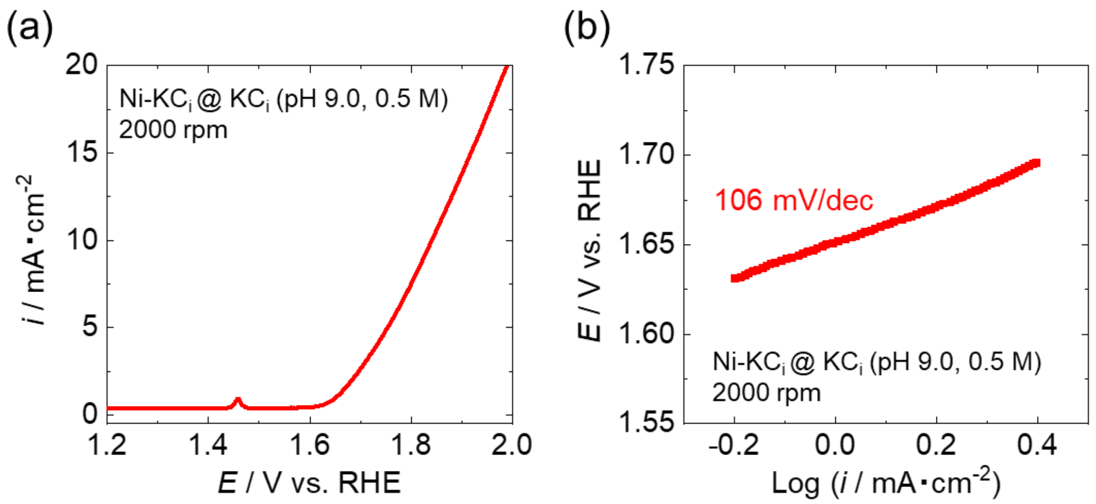

Figure S14. (a) LSV measurements of Ni-KC_i electrodes in 0.5 M KC_i (scan rate: 1 mV/s) and (b) the corresponding Tafel slope plots. In previous studies, the Tafel slope for the OER on pure NiOOH catalysts in borate buffer solution (pH 9.2) was reported to range between 90 and 120 mV/dec, indicating that this catalyst exhibits favorable activity as a Ni-based catalyst.[12]

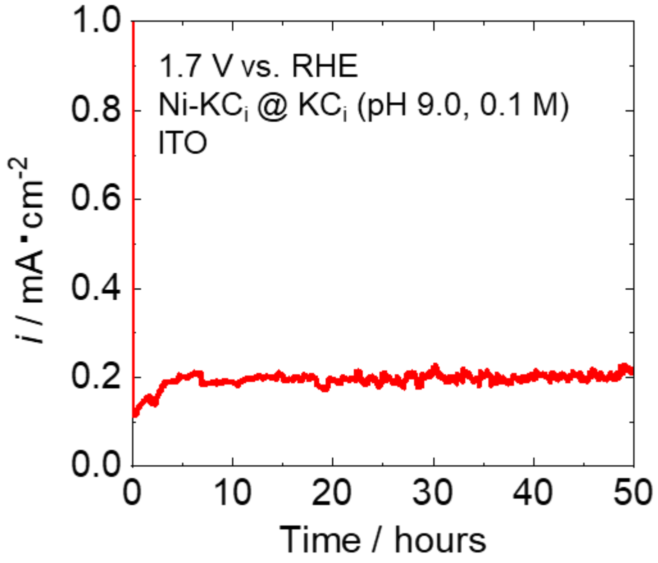

Figure S15. Long-term current–time measurement of the Ni-KC_i on an ITO substrate at 1.7 V vs. RHE in 0.1 M KC_i solution (pH 9.0), confirming that the catalyst maintains stable OER activity for over 50 hours.

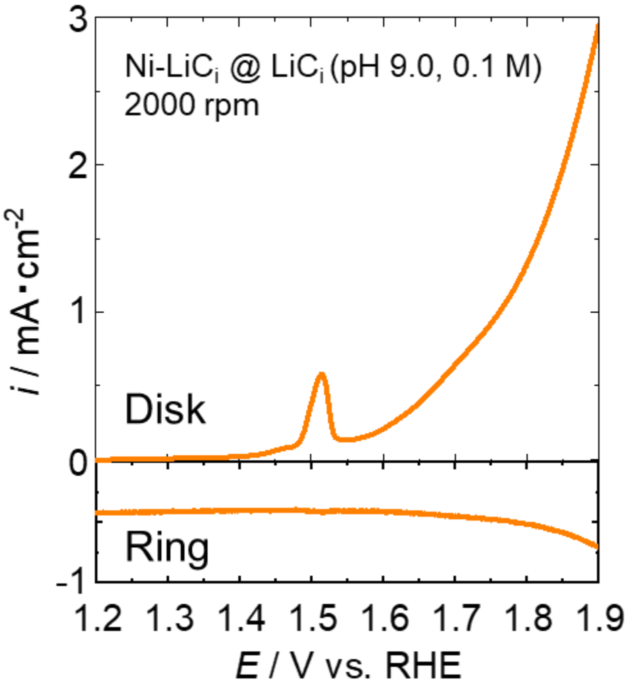

Figure S16. Electrochemical measurements for the prepared Ni-LiC_i electrodes on a Au rotating ring-disk electrode (RRDE) (2000 rpm) in 0.1 M LiC_i (pH 9.0): the upper panel shows a linear-sweep voltammogram of the oxygen evolution current measured at the Au disk electrode (scan rate: 1 mV/s); the lower panel shows the oxygen reduction current measured at the Pt ring electrode (held at 0.4 V vs. RHE). The RRDE working electrode consisted of a Au disk (diameter: 4 mm) and a Pt ring (inner diameter: 5 mm; outer diameter: 7 mm). The electrochemical measurements were controlled by a bipotentiostat (ALS/DY2323, BAS). These results clearly demonstrate that oxygen is generated by the Ni-LiC_i, as evidenced by the concurrent increase in both oxygen evolution and reduction currents observed beyond ~1.6 V.

v) *In-situ* UV-vis spectroscopic analysis

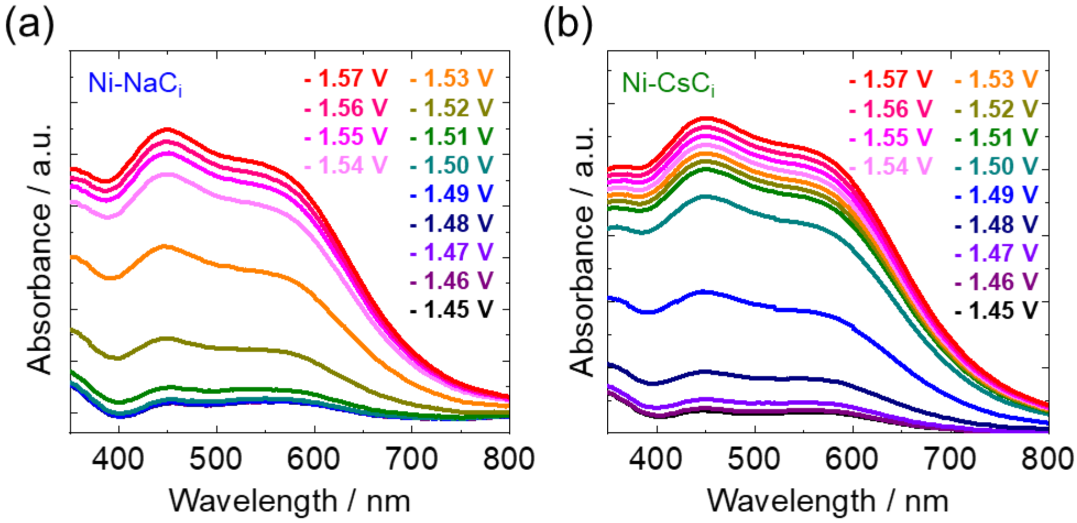

Figure S17. (a) *In situ* UV-vis spectra of Ni-*M*C$_i$ in *M*C$_i$ (pH 9.0): (a) Ni-NaC$_i$ and (b) Ni-CsC$_i$. The increase in absorbance upon potential stepping is attributed to electronic-structure changes associated with the Ni oxidation state. These results suggest that Cs^+ facilitates the oxidation of Ni^{2+} to higher-valent species (Ni^{3+}/Ni^{4+}) more effectively than Na^+, enabling the formation of active OER intermediates at lower potential.

vi) Operando Ni K-edge XAFS

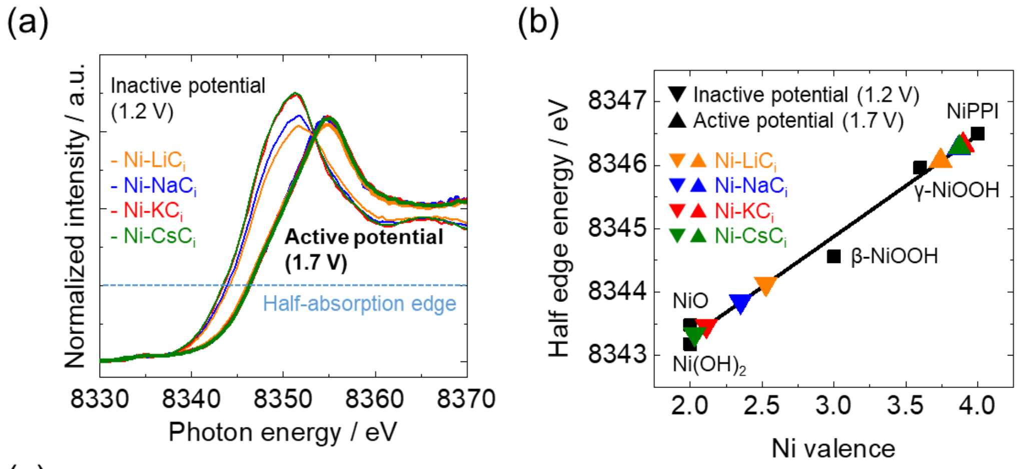

(c)

Ni-*M*C$_i$	Inactive potential (1.2 V)	Active potential (1.7 V)
Ni-LiC$_i$	2.5	3.7
Ni-NaC$_i$	2.4	3.9
Ni-KC$_i$	2.1	3.9
Ni-CsC$_i$	2.0	3.9

Figure S18. (a) Ni K-edge XANES spectra obtained from Ni-*M*C$_i$ (near the absorption edge). (b) Average Ni valence values as calculated from the relationship between Ni valence and the half-absorption edge (black line) and the position of the half-absorption edge in the spectrum of each Ni-*M*C$_i$. (c) Summary of the average Ni valence states. These data were acquired under inactive (1.2 V vs. RHE) and active (1.7 V vs. RHE) potential conditions. Orange, blue, red, and green correspond to Ni-LiC$_i$, Ni-NaC$_i$, Ni-KC$_i$, and Ni-CsC$_i$, respectively. These results indicate that, at the inactive potential (1.2 V vs. RHE), Ni-LiC$_i$ and Ni-NaC$_i$ retain relatively higher average Ni valence states than Ni-KC$_i$ and Ni-CsC$_i$, implying that Ni reduction is less complete in the presence of Li^+ and Na^+.

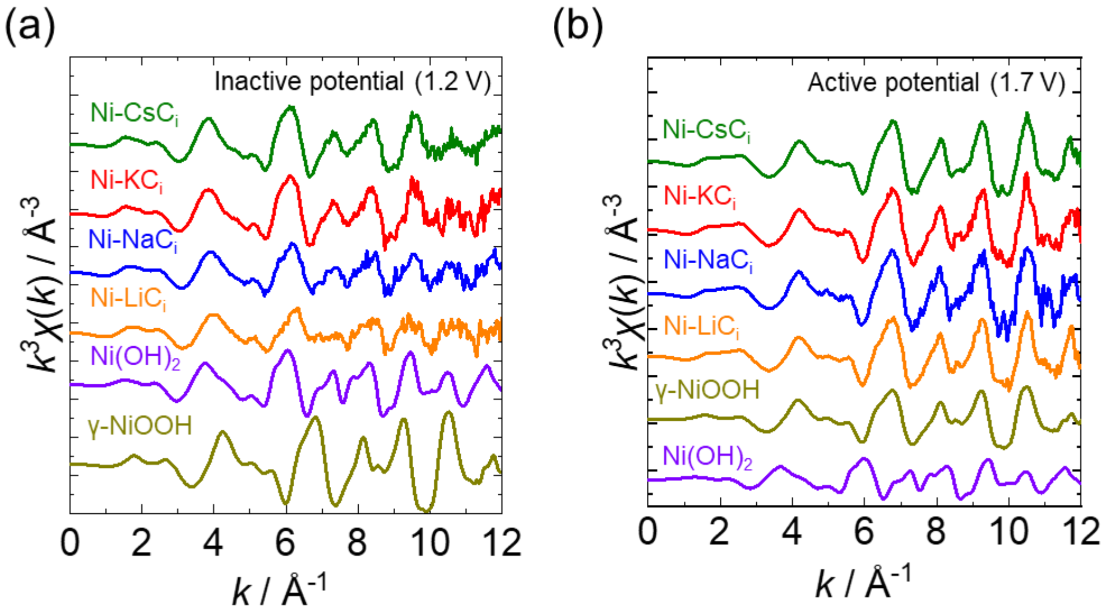

Figure S19. k^3-weighted Ni K-edge EXAFS spectra (a) inactive potential (1.2 V vs. RHE) and (b) active potential (1.7 V vs. RHE). The orange, blue, red, green, purple, and yellow-green lines correspond to Ni-LiC$_i$, Ni-NaC$_i$, Ni-KC$_i$, Ni-CsC$_i$, $Ni(OH)_2$, and γ-NiOOH respectively. These results indicate that all Ni-*M*C$_i$ electrodes measured in MC$_i$ electrolytes exhibit oscillation features consistent with γ-NiOOH at the active potential, whereas the oscillations are consistent with $Ni(OH)_2$ at the inactive potential; however, the amplitude around $k \approx 7$ Å^{-1} is attenuated in the presence of Li^+ and Na^+ and is almost completely canceled out under Li^+-containing conditions.

vii) Curve-fitting analysis of Ni K-edge FT-EXAFS spectra

(a)

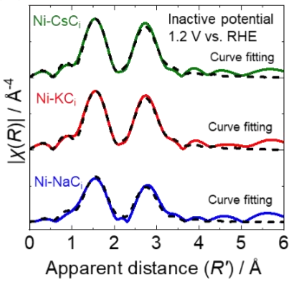

(b)

Ni-*M*C$_i$ (Inactive potential)	path	*N*	*R* (Å)	σ^2(Å^{-2})	ΔE^0 (eV)	R_f(%)
Ni-CsC$_i$ (1.2 V)	Ni-O	6	2.04(1)	0.006(1)	-2(1)	0.8
	Ni-Ni	6	3.08(1)	0.007(1)		
Ni-KC$_i$ (1.2 V)	Ni-O	6	2.04(1)	0.006(1)	-2(1)	0.6
	Ni-Ni	6	3.09(1)	0.007(1)		
Ni-NaC$_i$ (1.2 V)	Ni-O	6	2.04(2)	0.010(1)	0(2)	2.1
	Ni-Ni	6	3.10(2)	0.011(1)		

(c)

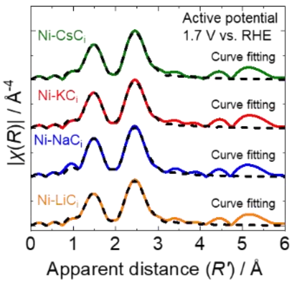

(d)

Ni-*M*C$_i$ (Active potential)	path	*N*	*R* (Å)	σ^2 (Å^{-2})	ΔE^0 (eV)	R_f(%)
Ni-CsC$_i$ (1.7 V)	Ni-O	6	1.89(1)	0.003(1)	-1(1)	0.4
	Ni-Ni	6	2.82(1)	0.003(1)		
Ni-KC$_i$ (1.7 V)	Ni-O	6	1.88(1)	0.003(1)	0(1)	0.3
	Ni-Ni	6	2.82(1)	0.003(1)		
Ni-NaC$_i$ (1.7 V)	Ni-O	6	1.89(1)	0.002(1)	0(1)	0.3
	Ni-Ni	6	2.82(1)	0.002(1)		
Ni-LiC$_i$ (1.7 V)	Ni-O	6	1.89(1)	0.004(1)	-1(1)	0.2
	Ni-Ni	6	2.82(1)	0.003(1)		

(e)

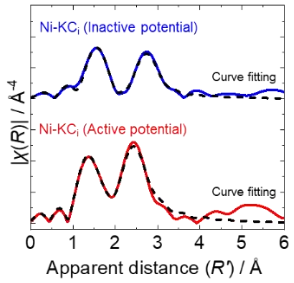

(f)

Ni-*M*C$_i$	path	*N*	*R* (Å)	σ^2(Å^{-2})	ΔE^0 (eV)	R_f(%)
Ni-KC$_i$ (1.2 V) (Inactive potential)	Ni-O	6	2.04(1)	0.006(1)	-2(1)	0.6
	Ni-Ni	6	3.09(1)	0.007(1)		
Ni-KC$_i$ (1.7 V) (Active potential)	Ni-O	6	1.88(1)	0.003(1)	-1(2)	0.4
	Ni-Ni	6	2.82(1)	0.003(1)		

Figure S20. (a) Curve-fitting analyses and (b) fitting parameters for Ni K-edge Fourier transforms of k^3-weighted EXAFS spectra of Ni-*M*C$_i$ at the inactive potential (1.2 V vs. RHE) ($3.0 \leq k \leq 9.3$, Hanning window function, not phase-shift corrected) and (c), (d) the active potential (1.7 V vs. RHE) ($3.0 \leq k \leq 11.0$, Hanning window function, not phase-shift corrected). (e) Curve-fitting analyses and (f) fitting parameters for the comparison of Ni K-edge Fourier transforms of k^3-weighted EXAFS spectra of Ni-KC$_i$ acquired at the inactive (1.2 V vs. RHE) and active (1.7 V vs. RHE) potentials ($3.0 \leq k \leq 9.3$, Hanning window function, not phase-shift corrected). R, σ^2, ΔE^0, and R_f are the bond length, Debye–Waller factor, edge-energy shift and reliability factor, respectively. During the fitting, the values of coordination number (N) were fixed at 6, and the amplitude reduction factor (S_0^2) were fixed at 0.80. These results indicate that the $Ni(OH)_2$ structures at the inactive potential were larger Debye-Waller factors in the presence of Na^+ than in the presence of K^+ or Cs^+. In contrast, the EXAFS fitting parameters for the γ-NiOOH phase at the active potential show no discernible dependence on the alkali-metal cation.

viii) Operando UV-vis spectroscopic analysis

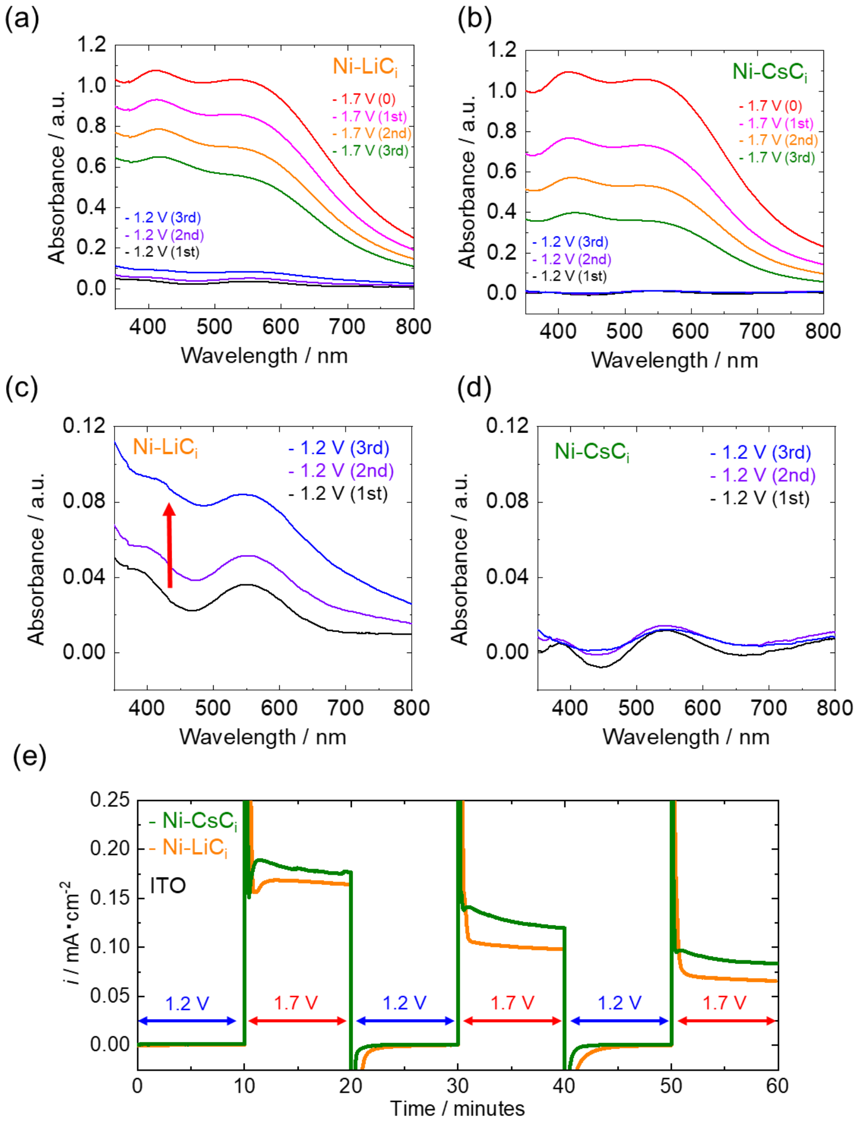

Figure S21. Operando UV-vis spectra for Ni-MC$_i$ in MC$_i$: (a) Ni-LiC$_i$ and (b) Ni-CsC$_i$ recorded at the inactive (1.2 V vs. RHE) and active (1.7 V vs. RHE) potentials during repeated potential cycling (1st–3rd cycles). Enlarged views of the spectra recorded at 1.2 V vs. RHE for (c) Ni-LiC$_i$ and (d) Ni-CsC$_i$. (e) Current–time measurement during potential switching between 1.2 and 1.7 V vs. RHE for Ni-LiC$_i$ (orange) and Ni-CsC$_i$ (green) on an ITO working electrode. These results indicate that repeated cycling increases the absorbance at 1.2 V vs. RHE for Ni-LiC$_i$, implying incomplete reversibility of the spectral changes in the presence of Li^+. In contrast, the current response during potential switching is consistently higher in the presence of Cs^+ than in the presence of Li^+.

ix) Operando Cs L(III)-edge XAFS spectra

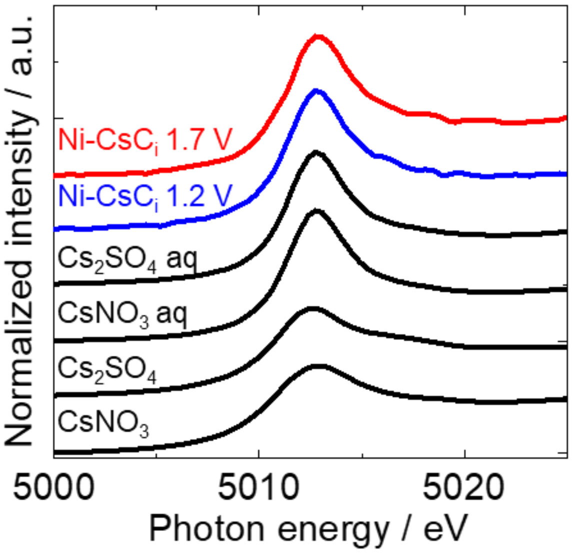

Figure S22. Operando Cs L(III)-edge XAFS spectra of Ni-CsC_i at 1.2 V (blue line) and 1.7 V (red line) potentials in CsC_i at pH 9.0. The spectra of Cs reference compounds are shown as black lines. The peak positions and spectral profiles of Ni-CsC_i closely resemble those of Cs_2SO_4 aq and $CsNO_3$ aq. This similarity indicates that the Cs species in the catalyst adopt a chemical state comparable to that of hydrated Cs^+ cations in aqueous solution. In addition, the interlayer Cs species within the catalyst are inferred to be hydrated by water molecules, existing as $Cs^+(H_2O)_y$.

References

[1] K. Yamada, T. Hiue, T. Ina, K. Wang, H. Kondoh, Y. Sakata, Y.-L. Lee, T. Kawai, M. Yoshida, "Improvement in Cobalt Phosphate Electrocatalyst Activity toward Oxygen Evolution from Water by Glycine Molecule Addition and Functional Details", *Anal. Sci.* **2020**, *36*, 35-39.

[2] A. Sakai, K. Harada, S. Tsunekawa, Y. Tamura, M. Ito, K. Hatada, T. Ina, T. Ohara, K.-H. Wang, T. Kawai, M. Yoshida, "Development of a MnCO3-based Electrocatalyst for Water Oxidation from Rhodochrosite Ore", *Chem. Lett.* **2022**, *51*, 723-727.

[3] T. Miura, S. Tsunekawa, S. Onishi, T. Ina, K. Wang, G. Watanabe, C. Hu, H. Kondoh, T. Kawai, M. Yoshida, "Assessing nickel oxide electrocatalysts incorporating diamines and having improved oxygen evolution activity using operando UV/visible and X-ray absorption spectroscopy", *Phys Chem Chem Phys* **2021**, *23*, 23280-23287.

[4] D. K. Bediako, B. Lassalle-Kaiser, Y. Surendranath, J. Yano, V. K. Yachandra, D. G. Nocera, "Structure-activity Correlations in a Nickel-borate Oxygen Evolution Catalyst", *J Am Chem Soc* **2012**, *134*, 6801-6809.

[5] K.-H. Wang, H. Ikeuchi, M. Yoshida, T. Miura, I. P. Liu, G. Watanabe, S. Cui, T. Kawai, "Nanometer-Thick Nickel Oxide Films Prepared from Alanine-Chelated Coordination Complexes for Electrochromic Smart Windows", *ACS Appl. Nano Mater.* **2020**, *3*, 9528-9537.

[6] H. Qin, Y. Yokoyama, Q. Fan, H. Iwatani, K. Tanaka, A. Sakaguchi, Y. Kanai, J. Zhu, Y. Onda, Y. Takahashi, "Investigation of cesium adsorption on soil and sediment samples from Fukushima Prefecture by sequential extraction and EXAFS technique", *Geochem. J.* **2012**, *46*, 297-302.

[7] K. I. Momma, F., "VESTA 3 for three-dimensional visualization of crystal, volumetric and morphology data", *J. Appl. Crystallogr.* **2011**, *44*, 1272–1276.

[8] B. Ravel, M. Newville, "ATHENA, ARTEMIS, HEPHAESTUS: Data Analysis for X-ray Absorption Spectroscopy using IFEFFIT", *J. Synchrotron Radiat.* **2005**, *12*, 537-541.

[9] M. Newville, "IFEFFIT : interactive XAFS Analysis and FEFF Fitting", *J. Synchrotron Radiat.* **2001**, *8*, 322-324.

[10] J. D. Michael, E. L. Demeter, S. M. Illes, Q. Fan, J. R. Boes, J. R. Kitchin, "Alkaline Electrolyte and Fe Impurity Effects on the Performance and Active-Phase Structure of NiOOH Thin Films for OER Catalysis Applications", *J. Phys. Chem. C* **2015**, *119*, 11475-11481.

[11] B. S. Yeo, A. T. Bell, "In Situ Raman Study of Nickel Oxide and Gold-Supported Nickel Oxide Catalysts for the Electrochemical Evolution of Oxygen", *J. Phys. Chem. C* **2012**,

116, 8394-8400.

[12] H. Xiao, H. Shin, W. A. Goddard, 3rd, "Synergy between Fe and Ni in the optimal performance of (Ni,Fe)OOH catalysts for the oxygen evolution reaction", *Proc Natl Acad Sci U S A* **2018**, *115*, 5872-5877.
